# Checklist of British and Irish Hymenoptera - Platygastroidea

**DOI:** 10.3897/BDJ.4.e7991

**Published:** 2016-04-22

**Authors:** Peter N. Buhl, Gavin R. Broad, David G. Notton

**Affiliations:** ‡ Troldhøjvej 3, Ølsted, Denmark; § The Natural History Museum, London, United Kingdom

**Keywords:** Platygastridae, Platygastrinae, Scelionidae, Sparasionidae, Scelioninae, Sceliotrachelinae, Teleasinae, Telenominae, fauna

## Abstract

**Background:**

A revised checklist of the British and Irish Platygastroidea (Platygastridae) substantially updates the previous comprehensive checklist, dating from 1978. Distribution data (i.e. occurrence in England, Scotland, Wales, Ireland and the Isle of Man) is reported where known.

**New information:**

A total of 381 British and Irish Platygastroidea represents a 47% increase on the number of British and Irish species reported in 1978.

## Introduction

This paper continues the series of checklists of the Hymenoptera of Britain and Ireland, starting with Broad and Livermore (2014a), Broad and Livermore (2014b) and Liston et al. (2014). The Introduction to the series (Broad 2014) sets out the background and rationale. Although there have been no comprehensive revisions of the majority of the platygastroid fauna there have been relatively recent catalogues of the Irish (O'Connor et al. 2004, O'Connor and Notton 2013) and British and Irish (Buhl and Notton 2009) faunas and a large body of taxonomic works describing and redescribing species that occur in the British Isles (e.g. Buhl 1995a, Buhl 1997, Buhl 2009b, Buhl 2009a, Buhl and Bennett 2009, Buhl and O'Connor 2010a, Buhl and O'Connor 2010c, Buhl and O'Connor 2011c, Mineo and O'Connor 2009, Notton 2006). The British and Irish list, at 381 confirmed species, is now 47% larger than in 1978 and there are probably many more species awaiting discovery.

**Figure 1.**
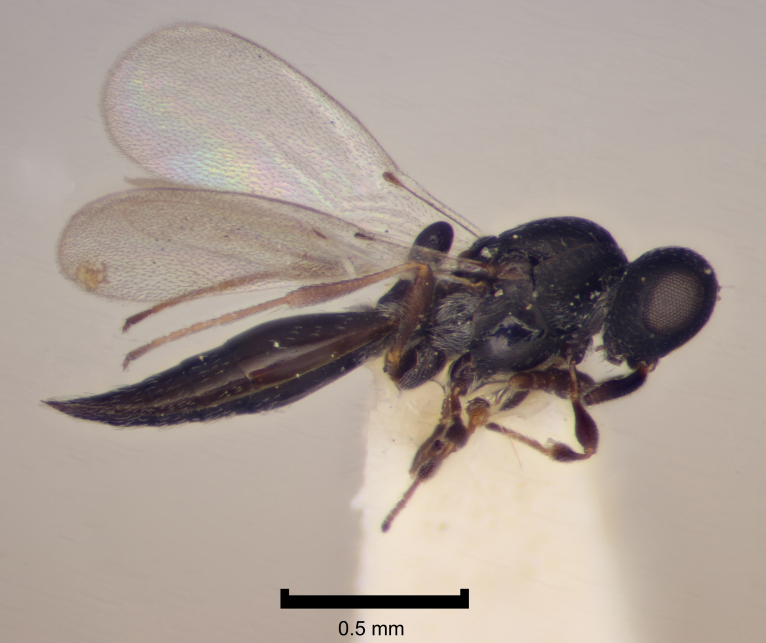
Representative British Platygastridae: Platygastrinae. *Inostemma mosellanae* Vlug det. D.G. Notton, female, England, Herts., Broadbalk, 1950, coll. H.F. Barnes, NHMUK010209581.

**Figure 2.**
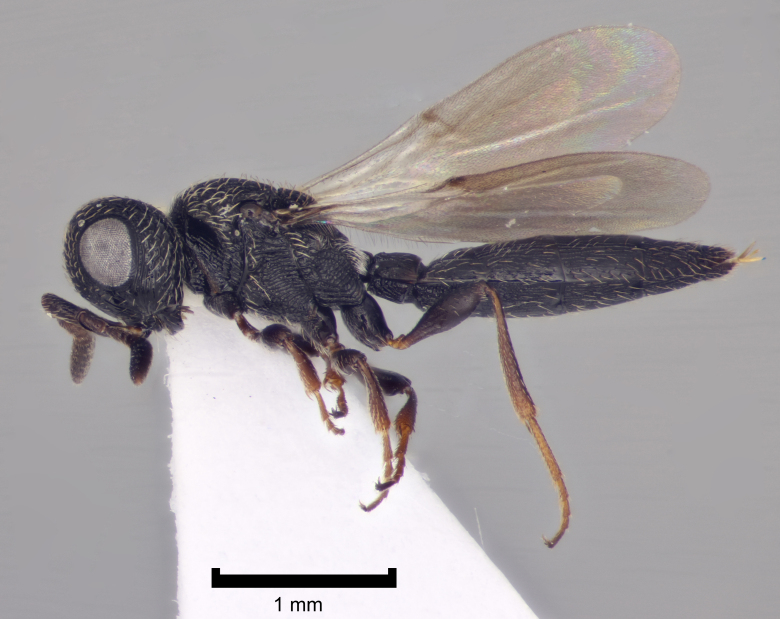
Representative British Scelionidae: Scelioninae. *Scelio rugulosus* Latreille det. A. Polaszek, female, England, Bucks., Ivinghoe Beacon SU963161, 22.8.2009, coll. A.M. George, NHMUK010209580.

Figs 1, 2 show some representative platygastroids. The Scelionidae (subfamilies Scelioninae, Teleasinae and Telenominae in Britain) and Sparasionidae comprise egg parasitoids, utilising a range of insects and spiders as hosts and acting as idiobiont endoparasitoids. In contrast, the vast majority of the Platygastridae (Platygastrinae and Sceliotrachelinae) are koinobiont egg-larval endoparasitoids of Cecidomyiidae gall midges (Diptera). A conspicuous exception is the genus *Amitus*, which are parasitoids of whiteflies (Hemiptera: Aleyrodidae) (Gauld and Bolton 1988 summarise the biology of Platygastroidea). As a consquence of their host ranges, platygastroids are generally small and rather neglected. Noyes et al. (1999) offer a fairly recent guide to the scattered identification literature. A number of species are used in biological control programmes (Orr 1988).

It has been recognised for some time that the traditional Scelionidae are paraphyletic with respect to the Platygastridae *s.s.* (e.g. Austin and Field 1997, Austin et al. 2005). A molecular phylogenetic study confirmed the paraphyly of ‘Scelionidae’ (Murphy et al. 2007) but the authors did not recommend taxonomic changes as the taxon sampling was limited. Despite this, Sharkey (2007) regarded the Platygastroidea as comprising one family, Platygastridae. This treatment has been followed, with caveats regarding the uncertainty in subfamily classification, in recent taxonomic works (e.g. Johnson et al. 2009, Talamas et al. 2009). In line with some other recent works (e.g. Ortega-Blanco et al. 2014, Talamas et al. 2015) we recognise Scelionidae as a separate family again, but recognising that some basal platygastroid groups previously classified as Scelionidae should be recognised at the family level, i.e. Nixoniidae and Sparasionidae, as Engel (2015) has also done. Such a classification system is much more informative than a single family for this biologically diverse superfamily, emphasising distinct biological differences between Platygastridae and the other families; maintains recognisable groups; acknowledges that the traditional Scelionidae is paraphyletic with respect to Playgastridae; and formally treating Sparasionidae - and the extralimital Nixoniidae - as separate families is congruent with the phylogenetic hypotheses presented so far.

## Materials and methods

Buhl and Notton (2009) revised the British and Irish checklist of Platygastridae and this forms the basis for the nomenclature and distribution data recorded here. Additional records from Buhl and O'Connor (2010a), Buhl and O'Connor (2010b), Buhl and O'Connor (2011b), Buhl and O'Connor (2012a), Buhl and O'Connor (2012b) and a few from P. Buhl. Dates of description and original combinations follow Vlug (1995). Nomenclature for Scelionidae mostly follows Johnson (1992)and the online Platygastroidea of the World, maintained by N.F. Johnson. Other useful works on these subfamilies include O'Connor et al. (2004), Notton (2006), O'Connor and Notton (2013). Synonymy post-Johnson (1992) and Vlug (1995) is referenced; we have tried to account for every name on the 1978 checklist (Fergusson 1978) and all additions to the fauna since then have been referenced. A more complete methodology can be found in Broad (2014). The following conventions and abbreviations are used here:

[***species***] taxon deleted from the British and Irish list

BMNH Natural History Museum, London

? status (including uncertain synonymy) or identification in the British Isles uncertain

misident. has been misidentified as this name

nom. dub. *nomen dubium*, a name of unknown or doubtful application

nom. ob. *nomen oblitum*, ‘forgotten name’, does not have priority over a younger name

nom. nov. *nomen novum*, a new replacement name

nom. nud. *nomen nudum*, an unavailable name

preocc. name preoccupied (junior homonym)

stat. rev. *status revocatus*, revived status (e.g., raised from synonymy)

unavailable not meeting the requirements of the International Code of Zoological Nomenclature

var. variety, only available as a valid name under the provisions of article 45.6 of the ICZN

Alternative versions of the checklist, as a formatted Word document and Excel spreadsheet, are provided here in the supplementary materials: Suppl. materials 1, 2.

Photographs were taken using a Canon EOS 450D digital camera attached to a Leica MZ12 stereomicroscope and partially focused images were combined using Helicon Focus v.4.80 software.

## Checklists

### Platygastridae

#### PLATYGASTROIDEA

Haliday, 1833

#### Platygastridae

Haliday, 1833

#### Platygastrinae

Haliday, 1833

#### Acerotella

Masner, 1964

#### Acerotella boter

(Walker, 1838)

Inostemma boter Walker, 1838

##### Distribution

Ireland, Isle of Man

#### Acerotella humilis

(Kieffer, 1913)

Acerota humilis Kieffer, 1913

##### Distribution

Ireland

##### Notes

added by Buhl and O'Connor (2012c)

#### Amblyaspis

Förster, 1856

#### Amblyaspis abas

(Walker, 1835)

Platygaster abas Walker, 1835

##### Distribution

England, Scotland

#### Amblyaspis belus

(Walker, 1835)

Platygaster belus Walker, 1835

##### Distribution

Ireland

#### Amblyaspis crates

(Walker, 1835)

Platygaster crates Walker, 1835

##### Distribution

England, Ireland, Isle of Man

#### Amblyaspis nereus

(Walker, 1835)

Platygaster nereus Walker, 1835

##### Distribution

England, Ireland

#### Amblyaspis otreus

(Walker, 1835)

Platygaster otreus Walker, 1835

##### Distribution

England, Scotland Ireland, Isle of Man

#### Amblyaspis prorsa

(Walker, 1835)

Platygaster prorsa Walker, 1835

##### Distribution

England, Scotland Ireland, Isle of Man

#### Amblyaspis roboris

(Haliday, 1835)

Platygaster roboris Haliday, 1835
lasiophila Kieffer, 1913

##### Distribution

England, Ireland, Isle of Man

#### Amblyaspis rufistilus

Kieffer, 1913

##### Distribution

Scotland

#### Amblyaspis rufithorax

Kieffer, 1913

##### Distribution

England

#### Amblyaspis rufiventris

Kieffer, 1913

##### Distribution

Scotland

#### Amblyaspis rufopetiolata

Kieffer, 1913

##### Distribution

England

##### Notes

PB regards *rufopetiolata* as a valid species, not a subspecies of *lasiophila*, although he has not seen the type.

#### Amblyaspis scelionoides

(Haliday, 1835)

Platygaster scelionoides Haliday, 1835
furius (Walker, 1835, *Platygaster*)
fuscicornis Thomson, 1859

##### Distribution

England, Ireland, Isle of Man

#### Amblyaspis scutellaris

Kieffer, 1904

##### Distribution

England

##### Notes

British specimens have been assigned to the subspecies *hyalina* Kieffer, 1914 (Buhl and Notton 2009).

#### Amblyaspis tritici

(Walker, 1835)

Platygaster tritici Walker, 1835

##### Distribution

England, Scotland, Ireland

#### Amblyaspis vestina

(Walker, 1835)

Platygaster vestinus Walker, 1835

##### Distribution

England

#### Amblyaspis vitellinipes

Kieffer, 1913

##### Distribution

Scotland

#### Anopedias

Förster, 1856

#### Anopedias lacustris

Kieffer, 1926

##### Distribution

England, Scotland, Wales, Ireland, Isle of Man

##### Notes

added by O'Connor et al. (2004)

#### Anopedias sundholmi

Huggert, 1974

##### Distribution

England

##### Notes

added by Buhl and Notton (2009)

#### Anopedias tritomus

Thomson, 1859

##### Distribution

England

##### Notes

added by Buhl and Notton (2009)​

#### Ceratacis

Thomson, 1859

#### Ceratacis cochleata

(Walker, 1835)

Platygaster cochleatus Walker, 1835
filicornis (Haliday, 1835, *Platygaster*)
munki (Buhl, 1994, *Platygaster*)

##### Distribution

England, Scotland, Wales, Ireland, Isle of Man

#### Ceratacis flavipes

Thomson, 1859

##### Distribution

England, Scotland, Ireland

##### Notes

added by Buhl and Notton (2009)​

#### Ceratacis laricis

(Haliday, 1835)

Platygaster laricis Haliday, 1835

##### Distribution

England, Scotland, Wales, Ireland

#### Euxestonotus

Fouts, 1925

XESTONOTUS Förster, 1856 preocc.
AXESTONOTUS Kieffer, 1926
EOXESTONOTUS Debauche, 1947

#### Euxestonotus clavicornis

Buhl, 1995

##### Distribution

England, Scotland

##### Notes

added by Buhl and Notton (2009)​

#### Euxestonotus error

(Fitch, 1861)

Platygaster error Fitch, 1861

##### Distribution

England, Scotland, Ireland, Isle of Man

##### Notes

Added by Milne (1960). Overlooked by Fergusson (1978) and by Buhl and Notton (2009).

#### Euxestonotus hasselbalchi

Buhl, 1995

##### Distribution

England, Wales, Ireland, Isle of Man

##### Notes

added by Buhl (1995b)

#### Euxestonotus parallelus

Kieffer, 1913

##### Distribution

Scotland

#### Gastrotrypes

Brues, 1922

#### Gastrotrypes spatulatus

Brues, 1922

##### Distribution

England

##### Notes

added by Buhl and Notton (2009)​

#### Inostemma

Haliday, 1833

PSILUS Jurine, 1807 preocc.
ACEROTA Förster, 1856
CERATOPSILUS Kieffer, 1913
BRACHINOSTEMMA Kieffer, 1916
BRACHYNOSTEMMA Risbec, 1953; incorrect subsequent spelling (Notton 2010)
INOCEROTA Szelényi, 1938

#### Inostemma boscii

(Jurine, 1807)

Psilus boscii Jurine, 1807

##### Distribution

Ireland, Isle of Man

#### Inostemma curtum

Szelényi, 1938

##### Distribution

England, Ireland

##### Notes

added by O'Connor et al. (2004)

#### Inostemma favo

Walker, 1838

##### Distribution

Ireland

#### Inostemma frivaldskyi

Szelényi, 1938

##### Distribution

Ireland

##### Notes

added by Buhl and O'Connor (2011b)

#### Inostemma hispo

Walker, 1838

##### Distribution

England, Ireland

#### Inostemma hyperici

Debauche, 1947

##### Distribution

Ireland

##### Notes

Added by O'Connor et al. (2004). Buhl and Notton (2009) record specimens tentatively identified as *hyperici* from England and Scotland.

#### Inostemma lycon

Walker, 1835

##### Distribution

England

#### Inostemma melicerta

Walker, 1835

##### Distribution

England, Ireland

#### Inostemma menippus

Walker, 1835

##### Distribution

England

#### Inostemma mosellanae

Vlug, 1991

##### Distribution

England

##### Notes

Added by Buhl and Notton (2009)​; Fig. 1.

#### Inostemma piricola

Kieffer, 1906

##### Distribution

England

##### Notes

Added by Barnes (1948). Omitted by Fergusson (1978).

#### Inostemma reticulatum

Szelényi, 1938

##### Distribution

England

##### Notes

added by Buhl and Notton (2009)​

#### Inostemma spinulosum

Kieffer, 1916

##### Distribution

England, Ireland

#### Inostemma walkeri

Kieffer, 1914

##### Distribution

England, Scotland, Ireland

#### Iphitrachelus

Haliday, 1835

#### Iphitrachelus gracilis

Masner, 1957

##### Distribution

England

#### Iphitrachelus lar

Haliday, 1835

##### Distribution

England, Scotland, Ireland, Isle of Man

#### Isocybus

Förster, 1856

#### Isocybus ascendens

Kieffer, 1913

##### Distribution

Scotland

#### Isocybus cameroni

Kieffer, 1913

##### Distribution

Scotland

#### Isocybus compressus

Kieffer, 1913

##### Distribution

Scotland

#### Isocybus cotta

(Walker, 1835)

Platygaster cotta Walker, 1835

##### Distribution

England, Scotland

#### Isocybus erato

(Walker, 1835)

Platygaster erato Walker, 1835

##### Distribution

England, Ireland

#### Isocybus grandis

(Nees, 1834)

Platygaster grandis Nees, 1834

##### Distribution

England

#### Isocybus horizontalis

Kieffer, 1913

##### Distribution

Scotland

#### Isocybus matuta

(Walker, 1835)

Platygaster matuta Walker, 1835

##### Distribution

England

#### Isocybus pyramidalis

Kieffer, 1913

##### Distribution

Scotland

#### Isocybus trochanteratus

Thomson, 1859

#### Isocybus walkeri

Kieffer, 1926

##### Distribution

England, Scotland, Ireland

#### Isostasius

Förster, 1856

MONOCRITA Förster, 1856
TRISINOSTEMMA Kieffer, 1914

#### Isostasius inserens

(Kirby, 1800)

Ichneumon inserens Kirby, 1800

##### Distribution

England

#### Isostasius punctiger

(Nees, 1834)

Platygaster punctiger Nees, 1834
atinus (Walker, 1835, *Inostemma*)
scrutator (Walker, 1835, *Inostemma*)

##### Distribution

England, Ireland

#### Isostasius sp.

##### Distribution

England, Wales

##### Notes

Recorded by Buhl and Notton (2009) as *Isostasius inserens* sensu Kozlov,1978, not *inserens* (Kirby, 1800).

#### Leptacis

Förster, 1856

MIRAMBLYASPIS Dodd, 1914
PROSAMBLYASPIS Kieffer, 1926

#### Leptacis ariadne

Buhl, 1999

##### Distribution

England

##### Notes

added by Buhl and Notton (2009)​

#### Leptacis coryphe

Buhl, 1998

##### Distribution

Ireland

##### Notes

aadded by Buhl and Notton (2009)​

#### Leptacis halia

(Walker, 1835)

Platygaster halia Walker, 1835

##### Distribution

England, Ireland

#### Leptacis laodice

(Walker, 1835)

Platygaster laodice Walker, 1835
buchi Buhl, 1997

##### Distribution

England, Ireland, Isle of Man

#### Leptacis lignicola

Kieffer, 1916

##### Distribution

England

##### Notes

added by Buhl and Notton (2009)​

#### Leptacis nice

(Walker, 1835)

Platygaster nice Walker, 1835

##### Distribution

England

#### Leptacis nydia

(Walker, 1835)

Platygaster nydia Walker, 1835
torispinula Huggert, 1980

##### Distribution

England

#### Leptacis orchymonti

(Debauche, 1947)

Anacoryphe orchymonti Debauche, 1947
Leptacis

##### Distribution

England, Wales, Ireland, Isle of Man

#### Leptacis ozines

(Walker, 1835)

Platygaster ozines Walker, 1835

##### Distribution

England, Wales, Ireland, Isle of Man

#### Leptacis tipulae

(Kirby, 1798)

Ichneumon tipulae Kirby, 1798
scutellaris (Nees, 1834, *Platygaster*)

##### Distribution

England, Ireland, Isle of Man

#### Leptacis tripartita

(Kieffer, 1913)

Amblyaspis tripartitus Kieffer, 1913

##### Distribution

Scotland

#### Leptacis vlugi

Buhl, 1997

##### Distribution

England, Ireland, Isle of Man

##### Notes

added by Buhl and Notton (2009)​

#### Metaclisis

Förster, 1856

PARINOSTEMMA Kieffer, 1914

#### Metaclisis areolata

(Haliday, 1835)

Inostemma areolata Haliday, 1835

##### Distribution

Ireland

#### Metaclisis montagnei

Maneval, 1936

##### Distribution

England, Scotland, Ireland

##### Notes

added by Buhl and Notton (2009)​

#### Metaclisis ocalea

(Walker, 1838)

Inostemma ocalea Walker, 1838

##### Distribution

England

#### Metaclisis phragmitis

Debauche, 1947

##### Distribution

England, Scotland, Wales

##### Notes

added by Buhl and Notton (2009)​

#### Metanopedias

Brues, 1910

DISYNOPEAS Kieffer, 1916

#### Metanopedias lasiopterae

(Kieffer, 1916)

Disynopeas lasiopterae Kieffer, 1916
britannicus Jackson, 1966

##### Distribution

England

#### Piestopleura

Förster, 1856

#### Piestopleura catillus

(Walker, 1835)

Platygaster catillus Walker, 1835

##### Distribution

England, Ireland

#### Piestopleura flavimanus

Kieffer, 1926

##### Distribution

England

#### Piestopleura mamertes

(Walker, 1835)

Platygaster mamertes Walker, 1835

##### Distribution

England, Ireland

#### Piestopleura seron

(Walker, 1835)

Platygaster seron Walker, 1835

##### Distribution

England, Ireland

#### Platygaster

Latreille, 1809

RHACODIA Panzer, 1838
HYPOCAMPSIS Förster, 1856
POLYGNOTUS Förster, 1856
ANEURHYNCHUS Provancher, 1887
COELOPELTA Ashmead, 1893
ANEURON Brues, 1910
PROSACTOGASTER Kieffer, 1914
TRIPLATYGASTER Kieffer, 1914
XESTONOTIDEA Gahan, 1919
PAREPIMECES Kieffer, 1926

#### Huggertella

Notton, 2006

CYLINDROGASTER Huggert, 1980

#### Platygaster (Huggertella) tubulosa

Brues, 1922

##### Distribution

England, Isle of Man

##### Notes

added by Notton (2006)

#### Parallelogaster

Huggert, 1973

#### Platygaster (Parallelogaster) lamelliformis

Huggert, 1973

##### Distribution

England, Scotland, Wales

##### Notes

added by Notton (2006)

#### Platygaster

Latreille, 1809

#### Platygaster (Platygaster) abia

Walker, 1835

##### Distribution

England, Scotland

#### Platygaster (Platygaster) abisares

Walker, 1835

cleodaeus Walker, 1835

##### Distribution

England, Ireland

#### Platygaster (Platygaster) abrupta

Buhl, 1994

##### Distribution

England

##### Notes

added by Buhl (1995b)

#### Platygaster (Platygaster) acrisius

Walker, 1835

##### Distribution

England, Ireland

#### Platygaster (Platygaster) aebeloeensis

Buhl, 2001

##### Distribution

England, Wales, Ireland, Isle of Man

##### Notes

added by Buhl and O'Connor (2009), Buhl and Bennett (2009)

#### Platygaster (Platygaster) aegeus

Walker, 1835

##### Distribution

England, Ireland, Isle of Man

#### Platygaster (Platygaster) anglica

Buhl, 2009

##### Distribution

England

##### Notes

added by Buhl (2009b)

#### Platygaster (Platygaster) ?anopediana

Buhl, 2005

##### Distribution

England

##### Notes

added by Buhl and Notton (2009)​

#### Platygaster (Platygaster) ashei

Buhl & O’Connor, 2012

##### Distribution

Ireland

##### Notes

added by Buhl and O'Connor (2012a)

#### Platygaster (Platygaster) athamas

Walker, 1835

##### Distribution

England, Scotland, Ireland

#### Platygaster (Platygaster) betulae

(Kieffer, 1916)

Misocyclops betulae Kieffer, 1916

##### Distribution

England, Wales, Ireland

##### Notes

added by Buhl and O'Connor (2009)

#### Platygaster (Platygaster) betularia

Kieffer, 1916

##### Distribution

England, Scotland, Ireland

##### Notes

added by Buhl and Notton (2009)​

#### Platygaster (Platygaster) breviscapa

Buhl, 2009

##### Distribution

England

##### Notes

added by Buhl and Notton (2009)​

#### Platygaster (Platygaster) bucolion

Walker, 1835

##### Distribution

England

#### Platygaster (Platygaster) cebes

Walker, 1835

cratinus Walker, 1835
olorus Walker, 1835

##### Distribution

England, Scotland, Wales, Isle of Man

#### Platygaster (Platygaster) cecidomyiae

Ratzeburg, 1852

##### Distribution

England

#### Platygaster (Platygaster) chloropus

Thomson, 1859

##### Distribution

England, Ireland

##### Notes

added by Buhl and Notton (2009)​

#### Platygaster (Platygaster) chrysippus

Walker, 1835

##### Distribution

England

#### Platygaster (Platygaster) clavata

Buhl, 1994

##### Distribution

Scotland, Wales

##### Notes

added by Buhl and Notton (2009)​

#### Platygaster (Platygaster) compressicornis

Thomson, 1859

pini (Kieffer, 1916, *Misocyclops*)
schlicki Buhl, 1995

##### Distribution

England, Scotland

##### Notes

added by Buhl (1995b)

#### Platygaster (Platygaster) confinis

Thomson, 1859

##### Distribution

Ireland

##### Notes

added by Buhl and O'Connor (2009)

#### Platygaster (Platygaster) consobrina

Kieffer, 1913

##### Distribution

Scotland

#### Platygaster (Platygaster) contorticornis

Ratzeburg, 1844

##### Distribution

England, Ireland

#### Platygaster (Platygaster) convergens

Kieffer, 1913

##### Distribution

Scotland

#### Platygaster (Platygaster) cottei

Kieffer, 1913

##### Distribution

Ireland

##### Notes

added by O'Connor et al. (2004)

#### Platygaster (Platygaster) cyrsilus

Walker, 1835

##### Distribution

England, Wales, Ireland

#### Platygaster (Platygaster) damokles

Buhl, 1998

##### Distribution

England

##### Notes

added by Buhl and Notton (2009)​

#### Platygaster (Platygaster) danica

Buhl, 1999

##### Distribution

Ireland

##### Notes

added by Buhl and O'Connor (2008)

#### Platygaster (Platygaster) deipyla

Walker, 1835

##### Distribution

England

#### Platygaster (Platygaster) demades

Walker, 1835

##### Distribution

England, Ireland, Isle of Man

#### Platygaster (Platygaster) dictys

Walker, 1835

##### Distribution

England

#### Platygaster (Platygaster) dryope

Walker, 1835

##### Distribution

Ireland, Isle of Man

#### Platygaster (Platygaster) elongata

Haliday, 1833

attenuata Walker, 1835
evadne Walker, 1835

##### Distribution

England, Wales, Ireland

#### Platygaster (Platygaster) enneatoma

(Kieffer, 1913)

Epimeces enneatomus Kieffer, 1913

##### Distribution

Scotland

#### Platygaster (Platygaster) ennius

Walker, 1835

##### Distribution

England, Scotland, Ireland

#### Platygaster (Platygaster) ensifer

(Westwood, 1833)

Epimeces ensifer Westwood, 1833

##### Distribution

England, Ireland

#### Platygaster (Platygaster) entwistlei

Buhl, 1997

##### Distribution

England, Scotland

##### Notes

added by Buhl (1997)

#### Platygaster (Platygaster) equestris

Spittler, 1969

##### Distribution

England

##### Notes

added by Buhl and Notton (2009)​

#### Platygaster (Platygaster) eriphyle

Walker, 1835

##### Distribution

England, Ireland

#### Platygaster (Platygaster) euhemerus

Walker, 1835

##### Distribution

England, Ireland, Isle of Man

#### Platygaster (Platygaster) floricola

(Kieffer, 1916)

Prosactogaster floricola Kieffer, 1916

##### Distribution

England, Ireland

##### Notes

added by Buhl and Notton (2009)​

#### Platygaster (Platygaster) frater

Buhl, 2006

##### Distribution

England, Ireland

##### Notes

added by Buhl and Notton (2009)​

#### Platygaster (Platygaster) galenus

Walker, 1835

##### Distribution

England, Ireland

#### Platygaster (Platygaster) germanica

Buhl, 1998

##### Distribution

England

##### Notes

added by Buhl and Notton (2009)​

#### Platygaster (Platygaster) gladiator

Zetterstedt, 1838

nitida Thomson, 1859

##### Distribution

England

##### Notes

added by Buhl and Notton (2009)​

#### Platygaster (Platygaster) gorge

Walker, 1835

##### Distribution

England

#### Platygaster (Platygaster) gracilipes

Huggert, 1975

##### Distribution

England, Scotland, Ireland, Isle of Man

##### Notes

added by Buhl (1995b)

#### Platygaster (Platygaster) gyge

Walker, 1835

longiventris Thomson, 1859

##### Distribution

England, Scotland, Ireland

#### Platygaster (Platygaster) henkvlugi

Buhl, 1996

##### Distribution

England, Scotland, Ireland

##### Notes

added by Buhl and O'Connor (2008)

#### Platygaster (Platygaster) herricki

Packard, 1841

##### Distribution

England

#### Platygaster (Platygaster) hibernica

Buhl & O'Connor, 2009

##### Distribution

Ireland

##### Notes

added by Buhl and O'Connor (2009)

#### Platygaster (Platygaster) hiemalis

Forbes, 1888

##### Distribution

England

##### Notes

Added by Gahan (1933); omitted by Fergusson (1978).

#### Platygaster (Platygaster) hybrida

Buhl, 1994

##### Distribution

England, Scotland

##### Notes

added by Buhl and Notton (2009)​

#### Platygaster (Platygaster) hyemalis

Curtis, 1830

#### Platygaster (Platygaster) inermis

Walker, 1835

##### Distribution

Ireland

##### Notes

added by Buhl and O'Connor (2008)

#### Platygaster (Platygaster) intermediana

Buhl, 2009

intermedia Buhl, 2006 preocc.

##### Distribution

England, Ireland

##### Notes

added by Buhl and O'Connor (2008)

#### Platygaster (Platygaster) iolas

Walker, 1835

##### Distribution

England

#### Platygaster (Platygaster) jutlandica

Buhl, 2006

##### Distribution

England

##### Notes

added by Buhl and Notton (2009)​

#### Platygaster (Platygaster) krarupi

Buhl, 1995

##### Distribution

England

##### Notes

added by Buhl and Notton (2009)​

#### Platygaster (Platygaster) leptines

Walker, 1835

##### Distribution

England, Ireland, Isle of Man

#### Platygaster (Platygaster) ?leucanthemi

(Kieffer, 1916)

Misocyclops leucanthemi Kieffer, 1916

##### Distribution

Wales

##### Notes

recorded by Buhl and Notton (2009)

#### Platygaster (Platygaster) ?lineata

Kieffer, 1906

##### Distribution

England

##### Notes

recorded by Barnes (1948)

#### Platygaster (Platygaster) lineaticeps

Buhl, 1994

##### Distribution

Scotland, Ireland

##### Notes

added by Buhl and Notton (2009)​

#### Platygaster (Platygaster) longestriolata

Thomson, 1859

##### Distribution

England, Ireland, Isle of Man

##### Notes

added by Buhl and O'Connor (2008)

#### Platygaster (Platygaster) longicaudata

Kieffer, 1906

##### Distribution

England

##### Notes

Added by Barnes (1927); omitted by Fergusson (1978).

#### Platygaster (Platygaster) lysicles

Walker, 1835

lativentris Thomson, 1859

##### Distribution

England, Scotland, Ireland, Isle of Man

#### Platygaster (Platygaster) malpighii

Kieffer, 1916

hanseni Buhl, 2006

##### Distribution

England, Ireland

##### Notes

added by Buhl and O'Connor (2008)

#### Platygaster (Platygaster) manensis

Buhl & Bennett, 2009

##### Distribution

Isle of Man

##### Notes

added by Buhl and Bennett (2009)

#### Platygaster (Platygaster) manto

Walker, 1835

##### Distribution

England, Scotland, Ireland, Isle of Man

#### Platygaster (Platygaster) marchali

Kieffer, 1906

##### Distribution

England

##### Notes

Added by Barnes (1931); omitted by Fergusson (1978).

#### Platygaster (Platygaster) marginata

Thomson, 1859

occipitalis Buhl, 1994

##### Distribution

England, Ireland

##### Notes

added by Buhl (1995b)

#### Platygaster (Platygaster) marshalli

(Kieffer, 1916)

Prosactogaster marshalli Kieffer, 1916

##### Distribution

England

#### Platygaster (Platygaster) martikaineni

Buhl, 2003

##### Distribution

England, Scotland

##### Notes

added by Buhl and Notton (2009)​

#### Platygaster (Platygaster) masneri

Huggert, 1975

##### Distribution

England, Ireland

##### Notes

added by Buhl and O'Connor (2008)

#### Platygaster (Platygaster) mayetiolae

Kieffer, 1916

##### Distribution

England

#### Platygaster (Platygaster) microsculpturata

Buhl, 1999

##### Distribution

England

##### Notes

aadded by Buhl and Notton (2009)​

#### Platygaster (Platygaster) minthe

Walker, 1835

laeviventris Thomson, 1859

##### Distribution

England

#### Platygaster (Platygaster) minutula

Dalla Torre, 1898

##### Distribution

England

##### Notes

Added by Barnes (1956); omitted by Fergusson (1978).

#### Platygaster (Platygaster) misella

Buhl, 2006

##### Distribution

England, Scotland

##### Notes

Added by Buhl and Notton (2009)​. Tentative identification (based on Scottish material) by Buhl and Notton (2009) but recorded, from reared English specimens, without any question marks by Buhl (2009a).

#### Platygaster (Platygaster) molsensis

Buhl, 1995

##### Distribution

England, Scotland, Wales

##### Notes

added by Buhl and Notton (2009)​

#### Platygaster (Platygaster) munita

Walker, 1835

##### Distribution

England, Wales, Ireland

#### Platygaster (Platygaster) nashi

Buhl & O’Connor, 2011

##### Distribution

Ireland

##### Notes

added Buhl and O'Connor (2011b)

#### Platygaster (Platygaster) nigra

Nees, 1834

nigerrimus (Kieffer, 1926, *Misocyclops*)

##### Distribution

Ireland

#### Platygaster (Platygaster) nisus

Walker, 1835

##### Distribution

England, Scotland, Wales, Ireland, Isle of Man

#### Platygaster (Platygaster) nixoni

Buhl, 2009

##### Distribution

England

##### Notes

added by Buhl (2009b)

#### Platygaster (Platygaster) oebalus

Walker, 1835

##### Distribution

England, Ireland, Isle of Man

#### Platygaster (Platygaster) oeclus

Walker, 1835

##### Distribution

England, Scotland, Ireland

#### Platygaster (Platygaster) orcus

Walker, 1835

##### Distribution

England, Ireland

#### Platygaster (Platygaster) orus

Walker, 1835

##### Distribution

England, Ireland

#### Platygaster (Platygaster) oscus

Walker, 1835

##### Distribution

England, Scotland, Ireland, Isle of Man

#### Platygaster (Platygaster) otanes

Walker, 1835

fuscipes Thomson, 1859

##### Distribution

England, Ireland, Isle of Man

#### Platygaster (Platygaster) pedasus

Walker, 1835

##### Distribution

England, Scotland, Ireland

#### Platygaster (Platygaster) pelias

Walker, 1835

apicalis Thomson, 1859
ruborum (Kieffer, 1916,)
crevecoeuri (Maneval, 1936, *Misocyclops*)

##### Distribution

England, Ireland, Isle of Man

##### Notes

Tentatively identified individuals recorded from Wales too (Buhl and Notton 2009).

#### Platygaster (Platygaster) philinna

Walker, 1835

nottoni Buhl, 1995

##### Distribution

England, Scotland, Wales, Ireland

##### Notes

English record from Buhl (1995c)

#### Platygaster (Platygaster) phragmitis

(Schrank, 1781)

Cynips phragmitis Schrank, 1781

#### Platygaster (Platygaster) picipes

Förster, 1861

##### Distribution

England

##### Notes

added by Buhl and Notton (2009)

#### Platygaster (Platygaster) pleuron

Walker, 1835

##### Distribution

England

#### Platygaster (Platygaster) plotina

Walker, 1835

##### Distribution

England

#### Platygaster (Platygaster) polita

Thomson, 1859

##### Distribution

Wales

##### Notes

added by Buhl and Notton (2009)​

#### Platygaster (Platygaster) puccinii

Vlug, 1995

nigripes Thomson, 1859 preocc.
thomsoni Buhl, 1995

##### Distribution

England, Wales, Ireland

##### Notes

added by Buhl and O'Connor (2008)

#### Platygaster (Platygaster) quadriceps

Buhl, 2006

##### Distribution

Ireland

##### Notes

added by Buhl and O'Connor (2010a)

#### Platygaster (Platygaster) quadrifaria

(Kieffer, 1916)

Polygnotus quadrifaria Kieffer, 1916

##### Distribution

England

#### Platygaster (Platygaster) rutilipes

Buhl, 1997

##### Distribution

England

##### Notes

added by Buhl and Notton (2009)​

#### Platygaster (Platygaster) rutubus

Walker, 1835

luteocoxalis (Kozlov, 1966, *Prosactogaster*)

##### Distribution

England

#### Platygaster (Platygaster) sagana

Walker, 1835

##### Distribution

England, Scotland, Wales, Ireland, Isle of Man

#### Platygaster (Platygaster) scotica

Kieffer, 1913

##### Distribution

Scotland

#### Platygaster (Platygaster) signata

(Förster, 1861)

Polygnotus signatus Förster, 1861

##### Distribution

Scotland, Ireland

##### Notes

added by Buhl and O'Connor (2009)

#### Platygaster (Platygaster) singularis

Buhl, 2006

##### Distribution

Ireland

##### Notes

added by Buhl and O'Connor (2009)

#### Platygaster (Platygaster) soederlundi

Buhl, 1998

##### Distribution

England

##### Notes

added by Buhl and Notton (2009)​

#### Platygaster (Platygaster) sonchis

Walker, 1835

##### Distribution

England

#### Platygaster (Platygaster) splendidula

Ruthe, 1859

leptocera Thomson, 1859
hirticornis Förster, 1861
lissonota Förster, 1861

##### Distribution

England, Ireland, Isle of Man

##### Notes

added by Buhl and O'Connor (2008)

#### Platygaster (Platygaster) sterope

Walker, 1835

##### Distribution

England, Isle of Man

#### Platygaster (Platygaster) strato

Walker, 1835

##### Distribution

Scotland

#### Platygaster (Platygaster) striatithorax

Buhl, 1994

##### Distribution

England, Wales

##### Notes

added by Buhl and Notton (2009)​

#### Platygaster (Platygaster) subapicalis

Buhl, 2006

##### Distribution

Ireland, Isle of Man

##### Notes

added by Buhl and Notton (2009)​, Buhl and O'Connor (2009)

#### Platygaster (Platygaster) sublongicornis

Buhl, 2009

##### Distribution

Scotland

##### Notes

added by Buhl (2009b)

#### Platygaster (Platygaster) subuliformis

(Kieffer, 1926)

Prosactogaster subuliformis Kieffer, 1926
Platygaster
subulatus misident.

##### Distribution

England, Ireland

##### Notes

added by Murchie et al. (1999)

#### Platygaster (Platygaster) suecica

(Kieffer, 1926)

Polygnotus suecicus Kieffer, 1926

##### Distribution

Ireland

##### Notes

added by Buhl and O'Connor (2008)

#### Platygaster (Platygaster) szelenyii

Huggert, 1975

crassus Szélenyi, 1958 preocc.

##### Distribution

England, Wales

##### Notes

added by Buhl and Notton (2009)​

#### Platygaster (Platygaster) taras

Walker, 1835

##### Distribution

England, Wales

#### Platygaster (Platygaster) tisias

Walker, 1835

siphon Förster, 1840

##### Distribution

England, Scotland, Wales, Ireland, Isle of Man

#### Platygaster (Platygaster) tuberata

Kieffer, 1926

tuberosa Kieffer, 1913 preocc.

##### Distribution

Scotland

#### Platygaster (Platygaster) tuberosula

Kieffer, 1926

##### Distribution

England

##### Notes

added by Buhl and Notton (2009)​

#### Platygaster (Platygaster) uniformis

Buhl, 2006

##### Distribution

England, Ireland

##### Notes

added by Buhl and Notton (2009)​

#### Platygaster (Platygaster) vaenia

Walker, 1835

ilione Walker, 1835

##### Distribution

England

#### Platygaster (Platygaster) virgo

Day, 1971

##### Distribution

England, Wales, Ireland

#### Platygaster (Platygaster) vulgaris

Buhl, 1998

##### Distribution

England

##### Notes

added by Buhl and Notton (2009)​

#### Platygaster (Platygaster) xeneus

Walker, 1838

##### Distribution

Ireland

#### Platygaster (Platygaster) zosine

Walker, 1835

##### Distribution

England

#### Urocyclops

Maneval, 1936

#### Platygaster (Urocyclops) depressiventris

Thomson, 1859

bettyae (Maneval, 1936, *Paracyclops*)
roosevelti (Debauche, 1947, *Urocyclops*)
humbolti (Fabritius & Grelimann, 1972, *Urocyclops*)

##### Distribution

England, Scotland, Wales

#### Synopeas

Förster, 1856

POLYMECUS Förster, 1856
DOLICHOTRYPES Crawford & Bradley, 1911

#### Synopeas aceris

Buhl & Bennett, 2009

##### Distribution

Isle of Man

##### Notes

added by Buhl and Bennett (2009)

#### Synopeas bohemani

Buhl, 1998

##### Distribution

England

##### Notes

added by Buhl and Notton (2009)​

#### Synopeas breve

Buhl, 1998

##### Distribution

Ireland, Isle of Man

##### Notes

BMNH, det. PB.; added by O'Connor et al. (2004)

#### Synopeas chica

Buhl, 2004

##### Distribution

England, Ireland

##### Notes

added by Buhl and Notton (2009)​

#### Synopeas ciliatum

Thomson, 1859

##### Distribution

England, Ireland, Isle of Man

#### Synopeas convexum

Thomson, 1859

##### Distribution

Ireland

##### Notes

Added by Buhl and O'Connor (2010a). Listed as Synopeas cf. convexum, occurring in England and Ireland, by Buhl and Notton (2009), then as a certain identification of Irish specimens by Buhl and O'Connor (2010a).

#### Synopeas craterus

(Walker, 1835)

Platygaster craterus Walker, 1835
gynomamertes (Hincks, 1944, *Ectadius*) invalid

##### Distribution

England, Ireland

#### Synopeas curvicauda

(Förster, 1856)

Sactogaster curvicauda Förster, 1856
Synopeas
longicauda (Förster, 1856, *Sactogaster*)
pisi (Förster, 1856, *Sactogaster*)

##### Distribution

England, Ireland, Isle of Man

#### Synopeas erinum

Buhl & O’Connor, 2010

##### Distribution

Ireland

##### Notes

added by Buhl and O'Connor (2010b)

#### Synopeas euryale

(Walker, 1835)

Platygaster euryale Walker, 1835

##### Distribution

England, Ireland

#### Synopeas fungorum

Buhl, 2000

##### Distribution

England

##### Notes

added by Buhl and Notton (2009)​

#### Synopeas fuscicola

Box, 1921

##### Distribution

England, Wales

#### Synopeas gallicola

Kieffer, 1916

##### Distribution

England

#### Synopeas gibberosum

Buhl, 1997

##### Distribution

England, Ireland

##### Notes

added by Buhl and O'Connor (2008)

#### Synopeas hibernicum

Buhl & O'Connor, 2009

##### Distribution

Ireland

##### Notes

added by Buhl and O'Connor (2009)

#### Synopeas hyllus

(Walker, 1835)

Platygaster hyllus Walker, 1835
figitiforme Thomson, 1859

##### Distribution

England, Scotland, Wales, Ireland

#### Synopeas inerme

Thomson, 1859

##### Distribution

England, Ireland

#### Synopeas jasius

(Walker, 1835)

Platygaster jasius Walker, 1835

##### Distribution

England, Ireland

#### Synopeas larides

(Walker, 1835)

Platygaster larides Walker, 1835

##### Distribution

England, Ireland

#### Synopeas latvianum

Buhl, 2009

##### Distribution

England, Isle of Man

##### Notes

added by Buhl and Bennett (2009)

#### Synopeas londiniense

Buhl, 2009

##### Distribution

England

##### Notes

added by Buhl (2009b)

#### Synopeas lugubre

Thomson, 1859

##### Distribution

England, Ireland

##### Notes

added by Notton (2008)

#### Synopeas manense

Buhl & Bennett, 2009

##### Distribution

Isle of Man

##### Notes

added by Buhl and Bennett (2009)

#### Synopeas myles

(Walker, 1835)

Platygaster myles Walker, 1835

##### Distribution

England, Scotland, Ireland, Isle of Man

#### Synopeas nottoni

Buhl, 2009

##### Distribution

England

##### Notes

added by Buhl (2009b)

#### Synopeas noyesi

Buhl, 2009

##### Distribution

England, Ireland

##### Notes

added by Buhl (2009b)

#### Synopeas opacum

Thomson, 1859

##### Distribution

England, Ireland

##### Notes

added by Buhl and O'Connor (2009)

#### Synopeas osaces

(Walker, 1835)

Platygaster osaces Walker, 1835

##### Distribution

England, Ireland

#### Synopeas rhanis

(Walker, 1835)

Platygaster rhanis Walker, 1835
acco (Walker, 1835, *Platygaster*)

##### Distribution

England, Ireland, Isle of Man

#### Synopeas robustum

Buhl, 2004

##### Distribution

England

##### Notes

added by Buhl and Notton (2009)​

#### Synopeas romsoeense

Buhl, 1997

##### Distribution

England, Isle of Man

##### Notes

added by Buhl and Bennett (2009)

#### Synopeas sosis

(Walker, 1835)

Platygaster sosis Walker, 1835
muticus misident.

##### Distribution

England, Scotland, Ireland, Isle of Man

##### Notes

*Synopeas muticus* (Nees, 1834, *Platygaster*) as recorded by Buhl and O'Connor (2008) refers to *sosis* (Buhl and O'Connor 2010b).

#### Synopeas tarsa

(Walker, 1835)

Platygaster tarsa Walker, 1835

##### Distribution

England, Ireland

#### Synopeas trebius

(Walker, 1835)

Platygaster trebius Walker, 1835

##### Distribution

England, Ireland, Isle of Man

#### Synopeas velutinum

(Walker, 1835)

Platygaster velutinus Walker, 1835

##### Distribution

England, Ireland

##### Notes

Described from English specimens, omitted by Fergusson (1978).

#### Synopeas ventrale

(Westwood, 1833)

Epimeces ventralis Westwood, 1833
abaris (Walker, 1835, *Platygaster*)

##### Distribution

England

#### Synopeas xanthopus

Kieffer, 1913

##### Distribution

Scotland

#### Trichacis

Förster, 1856

#### Trichacis didas

(Walker, 1835)

Platygaster didas Walker, 1835

##### Distribution

England, Ireland, Isle of Man

#### Trichacis pisis

(Walker, 1835)

Platygaster pisis Walker, 1835
opaca Thomson, 1859

##### Distribution

England, Ireland, Isle of Man

#### Trichacis remulus

(Walker, 1835)

Platygaster remulus Walker, 1835

##### Distribution

England

#### Sceliotrachelinae

Brues, 1908

#### Allotropa

Förster, 1856

EUROSTEMMA Szelényi, 1938
NASDIA Nixon, 1942
PLATYTROPA Kozlov, 1976

#### Allotropa europus

(Walker, 1838)

Inostemma europus Walker, 1838

##### Distribution

Ireland

#### Allotropa mecrida

(Walker, 1836)

Inostemma mecrida Walker, 1836

##### Distribution

England, Ireland, Isle of Man

#### Amitus

Haldeman, 1850

ZACRITA Förster, 1878
ELAPTUS Forbes, 1884

#### Amitus longicornis

(Förster, 1878)

Zacrita longicornis Förster, 1878

##### Distribution

England

##### Notes

added by Buhl and Notton (2009)

#### Amitus sp.

indet.

##### Distribution

England

##### Notes

*Amitus minervae* auctt., not *A. minervae* Silvestri; added by Polaszek (1997)

#### Fidiobia

Ashmead, 1894

ROSNETA Brues, 1908
TRICLAVUS Brèthes, 1916
FAHRINGERIA Kieffer, 1921
PLATYLLOTROPA Szelényi, 1938

#### Fidiobia hispanica

Popovici & Buhl, 2010

synergorum misident.

##### Distribution

Ireland

##### Notes

Added by Popovici and Buhl (2010) recorded as *Fidiobia synergorum* (Kieffer, 1921, *Fahringeria*) by O'Connor et al. (2004).

#### Platystasius

Nixon, 1937

ANOPEDIELLA Sundholm, 1956

#### Platystasius transversus

(Thomson, 1859)

Anopedias transversus Thomson, 1859
strangaliophagus Nixon, 1937

##### Distribution

England, Scotland, Ireland

### Scelionidae

#### Scelionidae

Haliday, 1839

##### Notes

Some Irish distribution data from O'Connor and Notton (2013).

#### Scelioninae

Haliday, 1839

#### Anteris

Förster, 1856

PARATRIMORUS Kieffer, 1908
TRICHACOLUS Kieffer, 1912

#### Anteris aethra

(Walker, 1836)

Telenomus aethra Walker, 1836

##### Distribution

England, Wales

##### Notes

Transferred from *Opisthacantha* by Notton (2006), who provides distribution data.

#### Anteris asramanes

(Walker, 1836)

Trimorus asramanes Walker, 1836
erdosi (Szabó, 1958, *Paratrimorus*)

##### Distribution

England, Ireland

##### Notes

Transferred from *Trimorus* by Notton (2006), who provides distribution data.

#### Baeus

Haliday, 1833

HYPERBAEUS Förster, 1856
PSILOBAEUS Kieffer, 1926

#### Baeus seminulum

Haliday, 1833

##### Distribution

England, Ireland

#### Eremioscelio

Priesner, 1951

#### Eremioscelio cydnoides

Priesner, 1951

##### Distribution

England

##### Notes

added by Notton (2006)

#### Gryon

Haliday, 1833

ACOLUS Förster, 1856
HADRONOTUS Förster, 1856
MUSCIDEA Motschoulsky, 1863
PLESIOBAEUS Kieffer, 1913

#### Gryon hospes

(Kieffer, 1913)

Plesiobaeus hospes Kieffer, 1913

#### Gryon misellum

Haliday, 1833

pumilio (Nees, 1834, *Teleas*)
divisus (Wollaston, 1858, *Telenomus*)
opacus (Thomson, 1859, *Acolus*)
basalis (Thomson, 1859, *Acolus*)
sagax (Kieffer, 1908, *Plastogryon*)
walkeri Kieffer, 1913

##### Distribution

Ireland

#### Idris

Förster, 1856

ACOLOIDES Howard, 1890
CERATOBAEUS Ashmead, 1893
PSEUDOBAEUS Perkins, 1910
DISSACOLUS Kieffer, 1926
MEGACOLUS Priesner, 1951

#### Idris flavicornis

Förster, 1856

krygeri (Kieffer, 1910, *Acolus*)

##### Distribution

England, Wales

##### Notes

added by Notton (2006)​

#### Macroteleia

Westwood, 1835

BAEONEURA Förster, 1856
PROSAPEGUS Kieffer, 1908
PARAPEGUS Kieffer, 1908

#### Macroteleia atrata

Kozloz & Kononova, 1987

##### Distribution

England

##### Notes

added by Notton (2006)​

#### Macroteleia bicolora

Kieffer, 1908

##### Distribution

England

##### Notes

added by Notton et al. (2014)

#### Macroteleia brevigaster

Masner, 1976

punctata (Kieffer, 1908, *Apegus*)
Macroteleia brevigaster ?*minor* Kozloz & Kononova, 1987

##### Distribution

England

##### Notes

added by Notton (2006)​

#### Psilanteris

Kieffer, 1916

OXYPHANURUS Kieffer, 1926

#### Psilanteris bicolor

(Kieffer, 1908)

Anteris bicolor Kieffer, 1908

##### Distribution

England, Wales

##### Notes

added by Notton (2006)​

#### Scelio

Latreille, 1805

ALERIA Marshall, 1874
CALOPTENOBIA Riley, 1878
ENNEASCELIO Kieffer, 1910

#### Scelio rugulosus

Latreille, 1805

#### Scelio vulgaris

Kieffer, 1908

##### Distribution

Ireland

##### Notes

added by O'Connor et al. (2004)

#### Scelio walkeri

Kieffer, 1913

#### Thoron

Haliday, 1833

NEOTHORON Masner, 1972

##### Notes

Distribution and synonymy from Johnson and Masner (2004).

#### Thoron metallicus

Haliday, 1833

fornicatus (Nees, 1834, *Teleas*)
solidus (Nees, 1834, *Teleas*)
gibbus Ruthe, 1859
nepea (Ferrière, 1916, *Anteris*)

##### Distribution

England, Ireland

#### Tiphodytes

Bradley, 1902

LIMNODYTES Marchal, 1900 preocc.
HUNGAROSCELIO Szabó, 1957

#### Tiphodytes gerriphagus

(Marchal, 1901)

Limnodytes gerriphagus Marchal, 1901
kaszabi (Szabó, 1957, *Hungaroscelio*)

##### Distribution

England, Ireland

##### Notes

added by Notton (2006)​

#### Teleasinae

Ashmead, 1893

#### Teleas

Latreille, 1809

PROTELEAS Kozlov, 1961

#### Teleas brasilas

Walker, 1836

#### Teleas clavicornis

(Latreille, 1805)

Scelio clavicornis Latreille, 1805
longicornis (Latreille, 1806, *Scelio*)

##### Distribution

Ireland

#### Teleas coriaceus

Kieffer, 1908

#### Teleas pulex

Walker, 1836

##### Distribution

Ireland

#### Teleas sibiricus

Kieffer, 1908

myrmecobius Kieffer, 1911

#### Trimorus

Förster, 1856

TRICHASIUS Provancher, 1887
PENTACANTHA Ashmead, 1888
HOPLOGRYON Ashmead, 1893
PARAGRYON Kieffer, 1908
ALLOGRYON Kieffer, 1910
HEMIMORUS Cameron, 1912
PROPENTACANTHA Kieffer, 1926
BRACHYSCELIO Risbec, 1950
PACHYSCELIO Risbec, 1954
SCUTELLIGRYON Szabó, 1966

#### Trimorus aegle

(Walker, 1836)

Teleas aegle Walker, 1836

##### Distribution

England, Scotland, Ireland

#### Trimorus algicola

(Kieffer, 1911)

Paragryon algicola Kieffer, 1911

#### Trimorus angustipennis

(Kieffer, 1908)

Hoplogryon angustipennis Kieffer, 1908

#### Trimorus antennalis

(Kieffer, 1908)

Hoplogryon antennalis Kieffer, 1908

##### Distribution

Scotland

#### Trimorus apricans

(Walker, 1836)

Teleas apricans Walker, 1836

##### Distribution

Ireland

#### Trimorus aratus

(Walker, 1836)

Teleas aratus Walker, 1836

##### Distribution

England, Wales, Ireland

#### Trimorus bacilliger

(Kieffer, 1908)

Hoplogryon bacilliger Kieffer, 1908

##### Distribution

Scotland, Ireland

##### Notes

Although described from Scotland by Kieffer (1908), listed as a synonym of *Trimorus pedestris* (Nees) (now placed in *Gryon*) by Fergusson (1978) until raised from synonymy by O'Connor and Mineo (2008).

#### Trimorus bassus

(Walker, 1836)

Teleas bassus Walker, 1836

#### Trimorus brevicollis

(Thomson, 1859)

Prosacantha brevicollis Thomson, 1859

#### Trimorus cameroni

(Kieffer, 1908)

Hoplogryon cameroni Kieffer, 1908

##### Distribution

Scotland

#### Trimorus carinatus

(Kieffer, 1908)

Hoplogryon carinatus Kieffer, 1908

#### Trimorus carinifrons

(Kieffer, 1908)

Hoplogryon carinifrons Kieffer, 1908

##### Distribution

Scotland

#### Trimorus cephisus

(Walker, 1836)

Teleas cephisus Walker, 1836

#### Trimorus chesias

(Walker, 1836)

Teleas chesias Walker, 1836

#### Trimorus chyllene

(Walker, 1836)

Teleas chyllene Walker, 1836

#### Trimorus elatior

(Walker, 1836)

Teleas elatior Walker, 1836

##### Distribution

England, Scotland, Ireland

#### Trimorus elongatus

(Kieffer, 1908)

Hoplogryon elongatus Kieffer, 1908

##### Distribution

England, Ireland

##### Notes

Although described from probable English material by Kieffer (1908), listed as a synonym of *puncticollis* by Fergusson (1978) but raised from synonymy by Kozlov (1978).

#### Trimorus ephippium

(Walker, 1836)

Teleas ephippium Walker, 1836

##### Distribution

England, Ireland

#### Trimorus flavipes

(Walker, 1836)

Teleas flavipes Walker, 1836
angustula (Thomson, 1859, *Prosacantha*)
rufipes (Thomson, 1859, *Prosacantha*)
similis (Thomson, 1859, *Prosacantha*)
cursor (Kieffer, 1908, *Hoplogryon*)
tardus (Kieffer, 1908, *Hoplogryon*)
nigerrimus (Kieffer, 1908, *Hoplogryon*)
rufimanus (Kieffer, 1908, *Hoplogryon*)
incompletus (Kieffer, 1908, *Hoplogryon*)
fimbriatus (Kieffer, 1908, *Hoplogryon*)

##### Distribution

England, Scotland, Ireland

#### Trimorus galba

(Walker, 1836)

Teleas galba Walker, 1836

#### Trimorus glaucus

(Walker, 1836)

Teleas glaucus Walker, 1836

#### Trimorus halteratus

(Kieffer, 1912)

Hoplogryon halterata Kieffer, 1912

#### Trimorus lamus

(Walker, 1836)

Teleas lamus Walker, 1836

##### Distribution

Ireland

#### Trimorus levigena

(Kieffer, 1908)

Hoplogryon levigena Kieffer, 1908

##### Distribution

Scotland

#### Trimorus lycaon

(Walker, 1836)

Teleas lycaon Walker, 1836

#### Trimorus marshalli

(Kieffer, 1913)

Hoplogryon marshalli Kieffer, 1913

#### Trimorus micropterus

(Kieffer, 1908)

Hoplogryon micropterus Kieffer, 1908

##### Distribution

Scotland

#### Trimorus myrmecobius

(Kieffer, 1911)

Hoplogryon myrmecobius Kieffer, 1911

#### Trimorus myrmecophilus

(Kieffer, 1911)

Paragryon myrmecophilus Kieffer, 1911

#### Trimorus nanno

(Walker, 1836)

Gryon nanno Walker, 1836

#### Trimorus nitidulus

(Thomson, 1859)

Prosacantha nitidula Thomson, 1859
pleuralis (Kieffer, 1908, *Hoplogryon*) preocc.
cursitans (Kieffer, 1908, *Hoplogryon*)
fulvimanus (Kieffer, 1908, *Hoplogryon*)
pleuricus (Kieffer, 1910, *Hoplogryon*)
bohemicus Masner, 1962

##### Distribution

England

#### Trimorus ocyroe

(Walker, 1836)

Teleas ocyroe Walker, 1836

#### Trimorus opacus

(Thomson, 1859)

Prosacantha opaca Thomson, 1859
pedestris misident.

##### Distribution

Ireland

#### Trimorus ovatus

(Thomson, 1859)

Prosacantha ovata Thomson, 1859
orbiculatus (Thomson, 1859, *Prosacantha*)
petiolaris (Thomson, 1859, *Prosacantha*)
rotundiventris (Thomson, 1859, *Prosacantha*)

#### Trimorus paula

(Walker, 1836)

Teleas paula Walker, 1836

##### Distribution

England, Wales, Ireland

#### Trimorus procris

(Walker, 1836)

Teleas procris Walker, 1836

##### Distribution

England, Ireland

#### Trimorus punctatifrons

(Kieffer, 1908)

Hoplogryon punctatifrons Kieffer, 1908

##### Distribution

Scotland

#### Trimorus puncticollis

(Thomson, 1859)

Prosacantha puncticollis Thomson, 1859
hylanipennis (Thomson, 1859, *Prosacantha*)
coxalis (Thomson, 1859, *Prosacantha*)

#### Trimorus rufonotatus

(Kieffer, 1908)

Hoplogryon rufonotatus Kieffer, 1908

##### Distribution

England, Scotland, Ireland

#### Trimorus sectigena

(Kieffer, 1908)

Hoplogryon sectigena Kieffer, 1908

##### Distribution

Ireland

##### Notes

added by O'Connor and Mineo (2008)

#### Trimorus striatigena

(Kieffer, 1908)

Hoplogryon striatigena Kieffer, 1908

##### Distribution

Scotland

#### Trimorus therycides

(Walker, 1836)

Teleas therycides Walker, 1836
doto (Walker, 1836, *Teleas*)
mermerus (Walker, 1836, *Teleas*)
smerdis (Walker, 1836, *Teleas*)
pallipes (Thomson, 1859, *Prosacantha*)
chloropus (Thomson, 1859, *Prosacantha*)
autumnalis (Thomson, 1859, *Prosacantha*)
brachyptera (Thomson, 1859, *Prosacantha*)
fuscimanus (Kieffer, 1908, *Hoplogryon*)
agilis (Kieffer, 1908, *Hoplogryon*)
microtomus (Kieffer, 1908, *Hoplogryon*)

##### Distribution

England, Scotland, Ireland

#### Trimorus timareta

(Walker, 1836)

Teleas timareta Walker, 1836

##### Distribution

England, Scotland, Ireland

#### Trimorus tuberculatus

(Kieffer, 1908)

Hoplogryon tuberculatus Kieffer, 1908

##### Distribution

Ireland

##### Notes

added by O'Connor and Mineo (2008)

#### Trimorus varicornis

(Walker, 1836)

Teleas varicornis Walker, 1836
metabus (Walker, 1836, *Teleas*)
minor (Thomson, 1859, *Prosacantha*)
grandis (Thomson, 1859, *Prosacantha*)
spinosa (Szépligeti, 1901, *Prosacantha*)
rufimanus (Kieffer, 1908, *Pentacantha*)

##### Distribution

England, Ireland

#### Trimorus xenetus

(Walker, 1836)

Teleas xenetus Walker, 1836

#### Xenomerus

Walker, 1836

NITEOGRYON Szabó, 1966

##### Notes

Distribution data and synonymy from Mikó et al. (2010).

#### Xenomerus canariensis

Huggert, 1974

hibernicus Mineo & O’Connor, 2009
mutator (Kononova & Kozlov, 2001, *Trimorus*)

##### Distribution

Ireland

##### Notes

added by Mineo and O'Connor (2009)

#### Xenomerus ergenna

Walker, 1836

medon (Walker, 1836, *Teleas*)
curtum (Kononova & Petrov, 1999, *Trimorus*)

##### Distribution

England, Ireland

#### Telenominae

Thomson, 1860

#### Telenomus

Haliday, 1833

HEMISIUS Westwood, 1833
PHANURUS Thomson, 1861
DISSOLCUS Ashmead, 1893
NEONECREMNUS Brèthes, 1909
ALLOPHANURUS Kieffer, 1912
HOMOPHANURUS Kieffer, 1912
PROPHANURUS Kieffer, 1912
LIOPHANURUS Kieffer, 1912
NEOTELENOMUS Dodd, 1913
AHOLCUS Kieffer, 1913
NANOPRIA Kieffer, 1913
NEOTELEIA Dodd, 1913
DISSOLCOIDES Dodd, 1913
PLATYTELENOMUS Dodd, 1914
PARIDRIS Brèthes, 1917
PSEUDOTELENOMUS Costa Lima, 1928
MICROMYMAR Risbec, 1950
APOROPHLEBUS Kozlov, 1970
PSEUDOPHANURUS Szabó, 1975
PSEUDOTELENOMOIDES Szabó, 1975
VERRUCOSICEPHALIA Szabó, 1975
ISSIDOTELENOMUS Pélev, 1975

##### Notes

Mineo et al. (2011) argue that *Verrucosicephalia* should be recognised as a separate genus, although N. Johnson *et al.*, in the online Platygastroidea catalogue, retain the name as a synonym of *Telenomus*.

#### Telenomus alcon

Walker, 1836

#### Telenomus andria

Walker, 1836

##### Distribution

Ireland

#### Telenomus ater

Haliday, 1833

#### Telenomus brachialis

Haliday, 1833

##### Distribution

Wales, Ireland

#### Telenomus cleostratus

Walker, 1836

#### Telenomus coilus

Walker, 1836

##### Distribution

Ireland

#### Telenomus colotes

Walker, 1836

#### Telenomus dalmanni

(Ratzeburg, 1844)

Teleas dalmanni Ratzeburg, 1844
orgyiae Fitch, 1865
fiskei Brues, 1910

##### Distribution

Ireland

#### Telenomus danubialis

(Szelényi, 1939)

Platytelenomus danubialis Szelényi, 1939
unilineatus (Szabó, 1975, *Platytelenomus*)

##### Distribution

England

##### Notes

added by Fergusson (1983a)

#### Telenomus depressus

(Szabó, 1975)

Verrucosicephalia depressa Szabó, 1975

##### Distribution

Ireland

##### Notes

Added by Mineo et al. (2011) as a species of *Verrucosicephalia*.

#### Telenomus dorsennus

Walker, 1836

#### Telenomus eris

Walker, 1836

##### Distribution

Ireland

#### Telenomus fergussoni

Buhl & O’Connor, 2012

##### Distribution

Ireland

##### Notes

added by Buhl and O'Connor (2012a)

#### Telenomus heliodorus

Mineo, 2006

##### Distribution

Ireland

##### Notes

added by O'Connor and Notton (2013)

#### Telenomus heteropterus

Haliday, 1833

nonnitens Szabó, 1978
pappi Szabó, 1978

##### Distribution

Ireland

#### Telenomus heydeni

Mayr, 1879

##### Distribution

Ireland

##### Notes

added by O'Connor and Mineo (2006)

#### Telenomus horus

Walker, 1836

#### Telenomus kolbei

Mayr, 1879

##### Distribution

Ireland

##### Notes

added by Buhl and O'Connor (2011a)

#### Telenomus laeviusculus

(Ratzeburg, 1844)

Teleas laeviusculus Ratzeburg, 1844

##### Distribution

Ireland

##### Notes

added by O'Connor and Mineo (2013)

#### Telenomus laricis

Walker, 1836

##### Distribution

Ireland

##### Notes

Listed by Mineo et al. (2011) as a species of *Verrucosicephalia*.

#### Telenomus longulus

Kozlov, 1967

##### Distribution

Ireland

##### Notes

added by O'Connor and Mineo (2009)

#### Telenomus lopicida

Silvestri, 1932

##### Distribution

Ireland

##### Notes

added by O'Connor and Mineo (2013)

#### Telenomus mentes

Walker, 1838

#### Telenomus minutus

(Westwood, 1833)

Hemisius minutus Westwood, 1833

#### Telenomus nauplius

Walker, 1836

#### Telenomus nitidulus

(Thomson, 1861)

Phanurus nitidulus Thomson, 1861
punctulatus (Ratzeburg, 1844, *Teleas*)
mayri (Kieffer, 1912, *Prophanurus*)

##### Distribution

Ireland

#### Telenomus orphne

Walker, 1836

#### Telenomus othonia

Walker, 1836

##### Distribution

England, Ireland

#### Telenomus othus

Haliday, 1833

##### Distribution

Ireland

#### Telenomus phalaenarum

(Nees, 1834)

Teleas phalaenarum Nees, 1834

#### Telenomus phylias

Walker, 1836

##### Distribution

Ireland

#### Telenomus pilumnus

Walker, 1836

#### Telenomus punctatissimus

(Ratzeburg, 1844)

Teleas punctatissimus Ratzeburg, 1844

#### Telenomus sitius

Walker, 1836

#### Telenomus stilpo

Walker, 1836

##### Distribution

Ireland

#### Telenomus tetratomus

(Thomson, 1861)

Phanurus tetratomus Thomson, 1861
bombycis Mayr, 1879
gracilis Mayr, 1879
verticillatus Kieffer, 1917

#### Telenomus tritia

Walker, 1836

#### Telenomus trophonius

Walker, 1836

#### Telenomus truncatus

(Nees, 1834)

Teleas truncatus Nees, 1834
linnei (Nees, 1834, *Teleas*)
zetterstedtii (Ratzeburg, 1844, *Teleas*)

#### Telenomus turesis

Walker, 1836

chloropus (Thomson, 1861, *Phanurus*); synonymy by Mineo et al. (2011)
sokolowi Mayr, 1897

##### Distribution

England, Ireland

#### Telenomus vibius

Walker, 1838

##### Distribution

England, Ireland

##### Notes

distribution data from Fergusson (1983b)

#### Telenomus vinicius

Walker, 1836

#### Trissolcus

Ashmead, 1893

ASOLCUS Nakagawa, 1900
APHANURUS Kieffer, 1912
IMMSIA Cameron, 1912
MICROPHANURUS Kieffer, 1926

#### Trissolcus arminon

(Walker, 1838)

Telenomus arminon Walker, 1838

##### Distribution

England

##### Notes

distribution data from Fergusson (1983b)

#### Trissolcus belenus

(Walker, 1836)

Telenomus belenus Walker, 1836

#### Trissolcus cultratus

(Mayr, 1879)

Telenomus cultratus Mayr, 1879
flavipes misident.

##### Distribution

England, ?Ireland

##### Notes

Added by Talamas et al. (2015); the Irish record of *Trissolcus flavipes* (Thomson, 1860, *Telenomus*) by O'Connor and Mineo (2007) probably refers to *cultratus*.

#### Trissolcus davatchii

(Javahery, 1968)

Asolcus davatchii Javahery, 1968

#### Trissolcus grandis

(Thomson, 1861)

Telenomus grandis Thomson, 1861
nigripes (Thomson, 1861, *Telenomus*)
frontalis (Thomson, 1861, *Telenomus*)
nigrita (Thomson, 1861, *Telenomus*)
nixomartini (Javahery, 1968, *Asolcus*)
silwoodensis (Javahery, 1968, *Asolcus*)

##### Distribution

England, Ireland

#### Trissolcus theste

(Walker, 1838)

Telenomus theste Walker, 1838

#### Trissolcus waloffae

(Javahery, 1968)

Asolcus waloffae Javahery, 1968

### Sparasionidae

#### Sparasionidae

Dahlbom, 1858

##### Notes

Nomenclature follows Johnson et al. (2008)

#### Sparasion

Latreille, 1802

OXYURUS Lamarck, 1817
BEBELUS Gistel, 1848
PROSPARASION Kieffer, 1913

#### Sparasion cephalotes

Latreille, 1802

frontalis Latreille, 1805

## Supplementary Material

Supplementary material 1 Checklist of British and Irish Platygastroidea Data type: spreadsheet
Brief description: Excel version of the Platygastroidea checklist
File: oo_85283.xlsx Buhl, P., Broad, G.R, and Notton, D.G.

Supplementary material 2 Checklist of British and Irish Platygastroidea Data type: text file
Brief description: alternative version of the Platygastroidea checklist, presented as a Word document.
File: oo_85301.docx Buhl, P., Broad, G.R. and Notton, D.G.
