# Supplementary material for: Checklist of British and Irish Hymenoptera - Platygastroidea
Source: Biodivers Data J. 2016 Apr 22;(4):e7991. doi: 10.3897/BDJ.4.e7991 (PMC4881931; doi:10.3897/BDJ.4.e7991)
Supplement: Supplementary material 2 — Checklist of British and Irish Platygastroidea [file biodiversity_data_journal-4-e7991-s002.docx]

Superfamily **Platygastroidea**

Peter Buhl, Gavin R. Broad & David G. Notton

It has been recognised for some time that the traditional Scelionidae are paraphyletic with respect to the Platygastridae *s. s.* (e.g. Austin & Field, 1997; Austin *et al.*, 2005). A recent molecular study confirmed the paraphyly of ‘Scelionidae’ (Murphy *et al.*, 2007) but the authors did not recommend taxonomic changes as the taxon sampling was limited. Despite this, Sharkey (2007) regarded the Platygastroidea as comprising one family, Platygastridae. This treatment has been followed, with caveats regarding the uncertainty in subfamily classification, in recent taxonomic works (e.g. Johnson *et al.*, 2009; Talamas *et al.*, 2009). In line with some recent works (e.g. Ortega-Blanco *et al.*, 2014; Talamas *et al.*, 2015) we recognise Scelionidae as a separate family again, but recognising that some basal platygastroid groups previously classified as Scelionidae should be recognised at the family level, i.e. Nixoniidae and Sparasionidae. Such a classification system is much more informative than a single family for this biologically diverse superfamily.

Family **Platygastridae** Haliday, 1833

Subfamily **PLATYGASTRINAE** Haliday, 1833^^[[1]](#footnote-2)^^

***Acerotella*** Masner, 1964

***boter*** (Walker, 1838, *Inostemma*) I M

***humilis*** (Kieffer, 1913, *Acerota*) I added by Buhl & O’Connor (2012*c*)

***Amblyaspis*** Förster, 1856

***abas*** (Walker, 1835, *Platygaster*) E S

***belus*** (Walker, 1835, *Platygaster*) I

***crates*** (Walker, 1835, *Platygaster*) E I M

***nereus*** (Walker, 1835, *Platygaster*) E I

***otreus*** (Walker, 1835, *Platygaster*) E S I M

***prorsa*** (Walker, 1835, *Platygaster*) E S I M

***roboris*** (Haliday, 1835, *Platygaster*) E I M

*lasiophila* Kieffer, 1913

***rufistilus*** Kieffer, 1913 S

***rufithorax*** Kieffer, 1913 E

***rufiventris*** Kieffer, 1913 S

***rufopetiolata*** Kieffer, 1913^^[[2]](#footnote-3)^^ E

***scelionoides*** (Haliday, 1835, *Platygaster*) E I M

*furius* (Walker, 1835, *Platygaster*)

*fuscicornis* Thomson, 1859

***scutellaris*** Kieffer, 1904 E^^[[3]](#footnote-4)^^

***tritici*** (Walker, 1835, *Platygaster*) E S I

***vestina*** (Walker, 1835, *Platygaster*) E

***vitellinipes*** Kieffer, 1913 S

***ANOPEDIAS*** Förster, 1856

***lacustris*** Kieffer, 1926 E S W I M added by O'Connor *et al.* (2004)

***sundholmi*** Huggert, 1974 E added by Buhl & Notton (2009)

***tritomus*** Thomson, 1859 E added by Buhl & Notton (2009)

***CERATACIS*** Thomson, 1859

***cochleata*** (Walker, 1835, *Platygaster*) E S W I M

*filicornis* (Haliday, 1835, *Platygaster*)

*munki* (Buhl, 1994, *Platygaster*)

***flavipes*** Thomson, 1859 E S I added by Buhl & Notton (2009)

***laricis*** (Haliday, 1835, *Platygaster*) E S W I

***EUXESTONOTUS*** Fouts, 1925

*XESTONOTUS* Förster, 1856 preocc.

*AXESTONOTUS* Kieffer, 1926

*EOXESTONOTUS* Debauche, 1947

***clavicornis*** Buhl, 1995 E S added by Buhl & Notton (2009)

***error*** (Fitch, 1861, *Platygaster*) E S I M added by Milne (1960)^^[[4]](#footnote-5)^^

***hasselbalchi*** Buhl, 1995 E W I M added by Buhl (1995*a*)

***parallelus*** Kieffer, 1913 S

***GASTROTRYPES*** Brues, 1922

***spatulatus*** Brues, 1922 E added by Buhl & Notton (2009)

***Inostemma*** Haliday, 1833

*PSILUS* Jurine, 1807 preocc.

*ACEROTA* Förster, 1856

*CERATOPSILUS* Kieffer, 1913

*CERASTOPSILUS* Kieffer, 1913 incorrect original spelling (Notton, 2010)

*BRACHINOSTEMMA* Kieffer, 1916

*BRACHYNOSTEMMA* Risbec, 1953 incorrect subsequent spelling (Notton, 2010)

*INOCEROTA* Szelényi, 1938

***boscii*** (Jurine, 1807, *Psilus*) I M

***curtum*** Szelényi, 1938 E I added by O'Connor *et al.* (2004)

***favo*** Walker, 1838 I

***frivaldskyi*** Szelényi, 1938 I added by Buhl & O’Connor (2011*b*)

***hispo*** Walker, 1838 E I

***hyperici*** Debauche, 1947 I added by O'Connor et al. (2004)^^[[5]](#footnote-6)^^

***lycon*** Walker, 1835 E

***melicerta*** Walker, 1835 E I

***menippus*** Walker, 1835 E

***mosellanae*** Vlug, 1991 E added by Buhl & Notton (2009)

***piricola*** Kieffer, 1906 E added by Barnes (1948)^^[[6]](#footnote-7)^^

***reticulatum*** Szelényi, 1938 E added by Buhl & Notton (2009)

***spinulosum*** Kieffer, 1916 E I

***walkeri*** Kieffer, 1914 E S I

***Iphitrachelus*** Haliday, 1835

***gracilis*** Masner, 1957 E

***lar*** Haliday, 1835 E S I M

***Isocybus*** Förster, 1856

***ascendens*** Kieffer, 1913 S

***cameroni*** Kieffer, 1913 S

***compressus*** Kieffer, 1913 S

***cotta*** (Walker, 1835, *Platygaster*) E S

***erato*** (Walker, 1835, *Platygaster*) E I

***grandis*** (Nees, 1834, *Platygaster*) E

***horizontalis*** Kieffer, 1913 S

***matuta*** (Walker, 1835, *Platygaster*) E

***pyramidalis*** Kieffer, 1913 S

***trochanteratus*** Thomson, 1859

***walkeri*** Kieffer, 1926 E S I

***Isostasius*** Förster, 1856

*MONOCRITA* Förster, 1856

*TRISINOSTEMMA* Kieffer, 1914

***inserens*** (Kirby, 1800, *Ichneumon*) E

***punctiger*** (Nees, 1834, *Platygaster*) E I

*atinus* (Walker, 1835, *Inostemma*)

*scrutator* (Walker, 1835, *Inostemma*)

**sp.** E W^^[[7]](#footnote-8)^^

***Leptacis*** Förster, 1856

*MIRAMBLYASPIS* Dodd, 1914

*PROSAMBLYASPIS* Kieffer, 1926

***ariadne*** Buhl, 1999 E added by Buhl & Notton (2009)

***coryphe*** Buhl, 1998 I added by Buhl & Notton (2009)

***halia*** (Walker, 1835, *Platygaster*) E I

***laodice*** (Walker, 1835, *Platygaster*) E I M

*buchi* Buhl, 1997

***lignicola*** Kieffer, 1916 E added by Buhl & Notton (2009)

***nice*** (Walker, 1835, *Platygaster*) E

***nydia*** (Walker, 1835, *Platygaster*) E

*torispinula* Huggert, 1980

***orchymonti*** (Debauche, 1947, *Anacoryphe*) E W I M

added by Buhl (1995*b*)

***ozines*** (Walker, 1835, *Platygaster*) E W I M

***tipulae*** (Kirby, 1798, *Ichneumon*) E I M

*scutellaris* (Nees, 1834, *Platygaster*)

***tripartita*** (Kieffer, 1913, *Amblyaspis*) S

***vlugi*** Buhl, 1997 E I M added by Buhl & Bennett (2009)

***Metaclisis*** Förster, 1856

*PARINOSTEMMA* Kieffer, 1914

***areolata*** (Haliday, 1835, *Inostemma*) I

***montagnei*** Maneval, 1936 E S I added by Buhl & Notton (2009)

***ocalea*** (Walker, 1838, *Inostemma*) E

***phragmitis*** Debauche, 1947 E S W added by Buhl & Notton (2009)

***Metanopedias*** Brues, 1910

*Disynopeas* Kieffer, 1916

***lasiopterae*** (Kieffer, 1916, *Disynopeas*) E

*britannicus* Jackson, 1966

***Piestopleura*** Förster, 1856

***catillus*** (Walker, 1835, *Platygaster*) E I

***flavimanus*** Kieffer, 1926 E

***mamertes*** (Walker, 1835, *Platygaster*) E I

***seron*** (Walker, 1835, *Platygaster*) E I

***Platygaster*** Latreille, 1809

*RHACODIA* Panzer, 1838 preocc.

*HYPOCAMPSIS* Förster, 1856

*POLYGNOTUS* Förster, 1856

*ANEURHYNCHUS* Provancher, 1887 preocc.

*COELOPELTA* Ashmead, 1893

*ANEURON* Brues, 1910

*PROSACTOGASTER* Kieffer, 1914

*TRIPLATYGASTER* Kieffer, 1914

*XESTONOTIDEA* Gahan, 1919

*PAREPIMECES* Kieffer, 1926

subgenus ***HUGGERTELLA*** Notton, 2006

*CYLINDROGASTER* Huggert, 1980 preocc.

***tubulosa*** Brues, 1922 E M^^[[8]](#footnote-9)^^ added by Notton (2006)

subgenus ***PARALLELOGASTER*** Huggert, 1973

***lamelliformis*** Huggert, 1973 E S W added by Notton (2006)

subgenus ***PLATYGASTER*** Latreille, 1809

***abia*** Walker, 1835 E S

***abisares*** Walker, 1835 E I

*cleodaeus* Walker, 1835

***abrupta*** Buhl, 1994 E added by Buhl (1995*b*)

***acrisius*** Walker, 1835 E I

***aebeloeensis*** Buhl, 2001 E W I M added by Buhl & O'Connor (2009), Buhl & Bennett (2009)

***aegeus*** Walker, 1835 E I M

***anglica*** Buhl, 2009 E added by Buhl (2009*a*)

**?*anopediana*** Buhl, 2005 E recorded by Buhl & Notton (2009)

***ashei*** Buhl & O’Connor, 2012 I added by Buhl & O’Connor (2012*a*)

***athamas*** Walker, 1835 E S I

***betulae*** (Kieffer, 1916, *Misocyclops*) E W I added by Buhl & O'Connor (2009)

***betularia*** Kieffer, 1916 E S I added by Buhl & Notton (2009)

***breviscapa*** Buhl, 2009 E added by Buhl & Notton (2009)

***bucolion*** Walker, 1835 E

***cebes*** Walker, 1835 E S W M

*cratinus* Walker, 1835

*olorus* Walker, 1835

***cecidomyiae*** Ratzeburg, 1852 E

***chloropus*** Thomson,1859 E I added by Buhl & O'Connor (2009)

***chrysippus*** Walker, 1835 E

***clavata*** Buhl, 1994 S W added by Buhl & Notton (2009)

***compressicornis*** Thomson, 1859 E S added by Buhl (1995*b*)

*pini* (Kieffer, 1916, *Misocyclops*)

*schlicki* Buhl, 1995

***confinis*** Thomson, 1859 I added by Buhl & O'Connor (2009)

***consobrina*** Kieffer, 1913 S

***contorticornis*** Ratzeburg, 1844 E I

***convergens*** Kieffer, 1913 S

***cottei*** Kieffer, 1913 I added by O'Connor *et al.* (2004)

***cyrsilus*** Walker, 1835 E W I

***damokles*** Buhl, 1998 E added by Buhl & Notton (2009)

***danica*** Buhl, 1999 I added by Buhl & O'Connor (2008)

***deipyla*** Walker, 1835 E

***demades*** Walker, 1835 E I M

***dictys*** Walker, 1835 E

***dryope*** Walker, 1835 I M

***elongata*** Haliday, 1833 E W I

*attenuata* Walker, 1835

*evadne* Walker, 1835

***enneatoma*** (Kieffer, 1913, *Epimeces*) S

***ennius*** Walker, 1835 E S I

***ensifer*** (Westwood, 1833, *Epimeces*) E I

***entwistlei*** Buhl, 1997 E S added by Buhl (1997)

***equestris*** Spittler, 1969 E added by Buhl & Notton (2009)

***eriphyle*** Walker, 1835 E I

***euhemerus*** Walker, 1835 E I M

***floricola*** (Kieffer, 1916, *Prosactogaster*) E I added by Buhl & Notton (2009)

***frater*** Buhl, 2006 E I added by Buhl & O'Connor (2009)

***galenus*** Walker, 1835 E I

***germanica*** Buhl, 1998 E added by Buhl & Notton (2009)

***gladiator*** Zetterstedt, 1838 E added by Buhl & Notton (2009)

*nitida* Thomson, 1859

***gorge*** Walker, 1835 E

***gracilipes*** Huggert, 1975 E S I M added by Buhl (1995*b*)

***gyge*** Walker, 1835 E S I

*longiventris* Thomson, 1859

***henkvlugi*** Buhl, 1996 E S I added by Buhl & O'Connor (2008)

***herricki*** Packard, 1841 E

***hibernica*** Buhl & O'Connor, 2009 I added by Buhl & O'Connor (2009)

***hiemalis*** Forbes, 1888 E added by Gahan (1933)^^[[9]](#footnote-10)^^

***hybrida*** Buhl, 1994 E S added by Buhl & Notton (2009)

***hyemalis*** Curtis, 1830

***inermis*** Walker, 1835 I added by Buhl & O'Connor (2008)

***intermediana*** Buhl, 2009 E I added by Buhl & O'Connor (2008)

*intermedia* Buhl, 2006 preocc.

***iolas*** Walker, 1835 E

***jutlandica*** Buhl, 2006 E added by Buhl & Notton (2009)

***krarupi*** Buhl, 1995 E added by Buhl & Notton (2009)

***leptines*** Walker, 1835 E I M

**?*leucanthemi*** (Kieffer, 1916, *Misocyclops*) W

recorded by Buhl & Notton (2009)

**?*lineata*** Kieffer, 1906 E recorded by Barnes (1948)

***lineaticeps*** Buhl, 1994 S I added by Buhl & Notton (2009)

***longestriolata*** Thomson, 1859 E I M added by Buhl & O'Connor (2008)

***longicaudata*** Kieffer, 1906 E added by Barnes (1927)^^[[10]](#footnote-11)^^

***lysicles*** Walker, 1835 E S I M

*lativentris* Thomson, 1859

***malpighii*** Kieffer, 1916 E I added by Buhl & O'Connor (2008)

*hanseni* Buhl, 2006 Buhl (2009*b*)

***manensis*** Buhl & Bennett, 2009 M added by Buhl & Bennett (2009)

***manto*** Walker, 1835 E S I M

***marchali*** Kieffer, 1906 E added by Barnes (1931)^^[[11]](#footnote-12)^^

***marginata*** Thomson, 1859 E I added by Buhl (1995*b*)

*occipitalis* Buhl, 1994

***marshalli*** (Kieffer, 1916, *Prosactogaster*) E

***martikaineni*** Buhl, 2003 E S added by Buhl & Notton (2009)

***masneri*** Huggert, 1975 E I added by Buhl & O'Connor (2008)

***mayetiolae*** Kieffer, 1916 E

***microsculpturata*** Buhl, 1999 E added by Buhl & Notton (2009)

***minthe*** Walker, 1835 E

*laeviventris* Thomson, 1859

***minutula*** Dalla Torre, 1898 E added by Barnes (1956)^^[[12]](#footnote-13)^^

***misella*** Buhl, 2006 E S added by Buhl & Notton (2009)^^[[13]](#footnote-14)^^

***molsensis*** Buhl, 1995 E S W added by Buhl & Notton (2009)

***munita*** Walker, 1835 E W I

***nashi*** Buhl & O’Connor, 2011 I added Buhl & O’Connor (2011*a*)

***nigra*** Nees, 1834 I

*nigerrimus* (Kieffer, 1926, *Misocyclops*)

***nisus*** Walker, 1835 E S W I M

***nixoni*** Buhl, 2009 E added by Buhl (2009*a*)

***oebalus*** Walker, 1835 E I M

***oeclus*** Walker, 1835 E S I

***orcus*** Walker, 1835 E I

***orus*** Walker, 1835 E I

***oscus*** Walker, 1835 E S I M

***otanes*** Walker, 1835 E I M

*fuscipes* Thomson, 1859

***pedasus*** Walker, 1835 E S I

***pelias*** Walker, 1835 E I M^^[[14]](#footnote-15)^^

*apicalis* Thomson, 1859

*ruborum* (Kieffer, 1916, *Misocyclops*)

*crevecoeuri* (Maneval, 1936, *Misocyclops*)

***philinna*** Walker, 1835 E^^[[15]](#footnote-16)^^ S W I

*nottoni* Buhl, 1995

***phragmitis*** (Schrank, 1781, *Cynips*)

***picipes*** Förster, 1861 E added by Buhl & Notton (2009)

***pleuron*** Walker, 1835 E

***plotina*** Walker, 1835 E

***polita*** Thomson, 1859 W added by Buhl & Notton (2009)

***puccinii*** Vlug, 1995 E W I added by Buhl & O'Connor (2008)

*nigripes* Thomson, 1859 preocc.

*thomsoni* Buhl, 1995

***quadriceps*** Buhl, 2006 I added by Buhl & O’Connor (2010a)

***quadrifaria*** (Kieffer, 1916), *Polygnotus* E

***rutilipes*** Buhl, 1997 E added by Buhl & Notton (2009)

***rutubus*** Walker, 1835 E

*luteocoxalis* (Kozlov, 1966, *Prosactogaster*)

***sagana*** Walker, 1835 E S W I M

***scotica*** Kieffer, 1913 S

***signata*** (Förster, 1861, *Polygnotus*) S I added by Buhl & O'Connor (2009)

***singularis*** Buhl, 2006 I added by Buhl & O'Connor (2009)

***soederlundi*** Buhl, 1998 E added by Buhl & Notton (2009)

***sonchis*** Walker, 1835 E

***splendidula*** Ruthe, 1859 E I M added by Buhl & O'Connor (2008)

*leptocera* Thomson, 1859

*hirticornis* Förster, 1861

*lissonota* Förster, 1861

***sterope*** Walker, 1835 E M

***strato*** Walker, 1835 S

***striatithorax*** Buhl, 1994 E W added by Buhl & Notton (2009)

***subapicalis*** Buhl, 2006 I M added by Buhl & Bennett (2009), Buhl & O'Connor (2009)

***sublongicornis*** Buhl, 2009 S added by Buhl (2009*a*)

***subuliformis*** (Kieffer, 1926, *Prosactogaster*) E I

added by Murchie *et al.* (1999)

*subulatus* misident.

***suecica*** (Kieffer, 1926, *Polygnotus*) I added by Buhl & O'Connor (2008)

***szelenyii*** Huggert, 1975 E W added by Buhl & Notton (2009)

*crassus* Szélenyi, 1958 preocc.

***taras*** Walker, 1835 E W

***tisias*** Walker, 1835 E S W I M

*siphon* Förster, 1840

***tuberata*** Kieffer, 1926 S

*tuberosa* Kieffer, 1913 preocc.

***tuberosula*** Kieffer, 1926 E added by Buhl & Notton (2009)

***uniformis*** Buhl, 2006 E I added by Buhl & Notton (2009)

***vaenia*** Walker, 1835 E

*ilione* Walker, 1835

***virgo*** Day, 1971 E W I

***vulgaris*** Buhl, 1998 E added by Buhl & Notton (2009)

***xeneus*** Walker, 1838 I

***zosine*** Walker, 1835 E

subgenus ***UROCYCLOPS*** Maneval, 1936

***depressiventris*** Thomson, 1859 E S W

*bettyae* (Maneval, 1936, *Paracyclops*)

*roosevelti* (Debauche, 1947, *Urocyclops*)

*humbolti* (Fabritius & Grelimann, 1972, *Urocyclops*)

***Synopeas*** Förster, 1856

*POLYMECUS* Förster, 1856

*DOLICHOTRYPES* Crawford & Bradley, 1911

***aceris*** Buhl & Bennett, 2009 M added by Buhl & Bennett (2009)

***bohemani*** Buhl, 1998 E added by Buhl & Notton (2009)

***breve*** Buhl, 1998 I M^^[[16]](#footnote-17)^^ added by O'Connor *et al.* (2004)

***chica*** Buhl, 2004 E I added by Buhl & Notton (2009)

***ciliatum*** Thomson, 1859 E I M

***convexum*** Thomson, 1859 I added by Buhl & O’Connor (2010*a*)^^[[17]](#footnote-18)^^

***craterus*** (Walker, 1835, *Platygaster*) E I

*mamertes* Kieffer, 1926

*gynomamertes* (Hincks, 1944, *Ectadius*) invalid

***curvicauda*** (Förster, 1856, *Sactogaster*) E I M

added by Buhl & Bennett (2009)

*longicauda* (Förster, 1856, *Sactogaster*)

*pisi* (Förster, 1856, *Sactogaster*)

***erinum*** Buhl & O’Connor, 2010 I added by Buhl & O’Connor (2010*b*)

***euryale*** (Walker, 1835, *Platygaster*) E I

***fungorum*** Buhl, 2000 E added by Buhl & Notton (2009)

***fuscicola*** Box, 1921 E W

***gallicola*** Kieffer, 1916 E

***gibberosum*** Buhl, 1997 E I added by Buhl & O'Connor (2008)

***hibernicum*** Buhl & O'Connor, 2009 I added by Buhl & O'Connor (2009)

***hyllus*** (Walker, 1835, *Platygaster*) E S W I

*figitiforme* Thomson, 1859

***inerme*** Thomson, 1859 E I

***jasius*** (Walker, 1835, *Platygaster*) E I

***larides*** (Walker, 1835, *Platygaster*) E I

***latvianum*** Buhl, 2009 E M added by Buhl & Bennett (2009)

***londiniense*** Buhl, 2009 E added by Buhl (2009*a*)

***lugubre*** Thomson, 1859 E I added by Notton (2008)

***manense*** Buhl & Bennett, 2009 M added by Buhl & Bennett (2009)

***myles*** (Walker, 1835, *Platygaster*) E S I M

***nottoni*** Buhl, 2009 E added by Buhl (2009*a*)

***noyesi*** Buhl, 2009 E I added by Buhl (2009*a*)

***opacum*** Thomson, 1859 E I added by Buhl & O'Connor (2009)

***osaces*** (Walker, 1835, *Platygaster*) E I

***rhanis*** (Walker, 1835, *Platygaster*) E I M

*acco* (Walker, 1835, *Platygaster*)

***robustum*** Buhl, 2004 E added by Buhl & Notton (2009)

***romsoeense*** Buhl, 1997 E M added by Buhl & Bennett (2009)

***sosis*** (Walker, 1835, *Platygaster*) E S I M

*muticus* misident.^^[[18]](#footnote-19)^^

***tarsa*** (Walker, 1835, *Platygaster*) E I

***trebius*** (Walker, 1835, *Platygaster*) E I M

***velutinum*** (Walker, 1835, *Platygaster*)^^[[19]](#footnote-20)^^ E I

***ventrale*** (Westwood, 1833, *Epimeces*) E

*abaris* (Walker, 1835, *Platygaster*)

***xanthopus*** Kieffer, 1913 S

***Trichacis*** Förster, 1856

***didas*** (Walker, 1835, *Platygaster*) E I M

***pisis*** (Walker, 1835, *Platygaster*) E I M

*opaca* Thomson, 1859

***remulus*** (Walker, 1835, *Platygaster*) E

Subfamily **Sceliotrachelinae** Brues, 1908

***Allotropa*** Förster, 1856

*EUROSTEMMA* Szelényi, 1938

*NASDIA* Nixon, 1942

*PLATYTROPA* Kozlov, 1976

***europus*** (Walker, 1838, *Inostemma*) I

***mecrida*** (Walker, 1836, *Inostemma*) E I M

***Amitus*** Haldeman, 1850

*ZACRITA* Förster, 1878

*ELAPTUS* Forbes, 1884

***longicornis*** (Förster, 1878, *Zacrita*) E added by Buhl & Notton (2009)

**sp. indet.**^^[[20]](#footnote-21)^^ E added by Polaszek (1997)

***FIDIOBIA*** Ashmead, 1894

*ROSNETA* Brues, 1908

*TRICLAVUS* Brèthes, 1916

*FAHRINGERIA* Kieffer, 1921

*PLATYLLOTROPA* Szelényi, 1938

***hispanica*** Popovici & Buhl, 2010 I added by Popovici & Buhl (2010)

*synergorum* misident.^^[[21]](#footnote-22)^^ added by O'Connor *et al.* (2004)

***Platystasius*** Nixon, 1937

*ANOPEDIELLA* Sundholm, 1956

***transversus*** (Thomson, 1859, *Anopedias*) E S I

*strangaliophagus* Nixon, 1937

Family **Scelionidae** Haliday, 1839**^^[[22]](#footnote-23)^^**

Subfamily **Scelioninae** Haliday, 1839

***ANTERIS*** Förster, 1856

*PARATRIMORUS* Kieffer, 1908

*TRICHACOLUS* Kieffer, 1912

***aethra*** (Walker, 1836, *Telenomus*)^^[[23]](#footnote-24)^^ E W

***asramanes*** (Walker, 1836, *Trimorus*)^^[[24]](#footnote-25)^^ E I

*erdosi* (Szabó, 1958, *Paratrimorus*) Notton (2006)

***Baeus*** Haliday, 1833

*HYPERBAEUS* Förster, 1856

*PSILOBAEUS* Kieffer, 1926

***seminulum*** Haliday, 1833 E I

***EREMIOSCELIO*** Priesner, 1951

***cydnoides*** Priesner, 1951 E added by Notton (2006)

***Gryon*** Haliday, 1833

*ACOLUS* Förster, 1856

*HADRONOTUS* Förster, 1856

*MUSCIDEA* Motschoulsky, 1863

*Plesiobaeus* Kieffer, 1913

***hospes*** (Kieffer, 1913, *Plesiobaeus*)

***misellum*** Haliday, 1833 I

*pumilio* (Nees, 1834, *Teleas*)

*divisus* (Wollaston, 1858, *Telenomus*)

*opacus* (Thomson, 1859, *Acolus*)

*basalis* (Thomson, 1859, *Acolus*)

*sagax* (Kieffer, 1908, *Plastogryon*)

*walkeri* Kieffer, 1913

***IDRIS*** Förster, 1856

*ACOLOIDES* Howard, 1890

*CERATOBAEUS* Ashmead, 1893

*PSEUDOBAEUS* Perkins, 1910

*DISSACOLUS* Kieffer, 1926

*MEGACOLUS* Priesner, 1951

***flavicornis*** Förster, 1856 E W added by Notton (2006)

*krygeri* (Kieffer, 1910, *Acolus*)

***MACROTELEIA*** Westwood, 1835

*BAEONEURA* Förster, 1856

*PROSAPEGUS* Kieffer, 1908

*PARAPEGUS* Kieffer, 1908

***atrata*** Kozloz & Kononova, 1987 E added by Notton (2006)

***bicolora*** Kieffer, 1908 E added by Notton *et al.* (2014)

***brevigaster*** Masner, 1976 E added by Notton (2006)

*punctata* (Kieffer, 1908, *Apegus*) preocc.

?*minor* Kozloz & Kononova, 1987 E Notton (2006)

***PSILANTERIS*** Kieffer, 1916

*OXYPHANURUS* Kieffer, 1926

***bicolor*** (Kieffer, 1908, *Anteris*) E W added by Notton (2006)

***Scelio*** Latreille, 1805

*ALERIA* Marshall, 1874

*CALOPTENOBIA* Riley, 1878

*ENNEASCELIO* Kieffer, 1910

***rugulosus*** Latreille, 1805 E

***vulgaris*** Kieffer, 1908 I added by O’Connor *et al.* (2004)

***walkeri*** Kieffer, 1913

***Thoron*** Haliday, 1833^^[[25]](#footnote-26)^^

*Neothoron* Masner, 1972

***metallicus*** Haliday, 1833 E I

*fornicatus* (Nees, 1834, *Teleas*)

*solidus* (Nees, 1834, *Teleas*)

*gibbus* Ruthe, 1859

*nepea* (Ferrière, 1916, *Anteris*)

***TIPHODYTES*** Bradley, 1902

*LIMNODYTES* Marchal, 1900 preocc.

*HUNGAROSCELIO* Szabó, 1957

***gerriphagus*** (Marchal, 1901, *Limnodytes*) E I added by Notton (2006)

*kaszabi* (Szabó, 1957, *Hungaroscelio*)

Subfamily **Teleasinae** Ashmead, 1893

***Teleas*** Latreille, 1809

*PROTELEAS* Kozlov, 1961

***brasilas*** Walker, 1836

***clavicornis*** (Latreille, 1805, *Scelio*) I

*longicornis* (Latreille, 1806, *Scelio*)

***coriaceus*** Kieffer, 1908

***pulex*** Walker, 1836 I

***sibiricus*** Kieffer, 1908

*myrmecobius* Kieffer, 1911

***Trimorus*** Förster, 1856

*TRICHASIUS* Provancher, 1887

*PENTACANTHA* Ashmead, 1888

*HOPLOGRYON* Ashmead, 1893

*PARAGRYON* Kieffer, 1908

*ALLOGRYON* Kieffer, 1910

*HEMIMORUS* Cameron, 1912

*PROPENTACANTHA* Kieffer, 1926

*BRACHYSCELIO* Risbec, 1950 preocc.

*PACHYSCELIO* Risbec, 1954

*SCUTELLIGRYON* Szabó, 1966

***aegle*** (Walker, 1836, *Teleas*) E S I

***algicola*** (Kieffer, 1911, *Paragryon*)

***angustipennis*** (Kieffer, 1908, *Hoplogryon*)

***antennalis*** (Kieffer, 1908, *Hoplogryon*) S

***apricans*** (Walker, 1836, *Teleas*) I

***aratus*** (Walker, 1836, *Teleas*) E W I

***bacilliger*** (Kieffer, 1908, *Hoplogryon*) S I^^[[26]](#footnote-27)^^

***bassus*** (Walker, 1836, *Teleas*)

***brevicollis*** (Thomson, 1859, *Prosacantha*)

***cameroni*** (Kieffer, 1908, *Hoplogryon*) S

***carinatus*** (Kieffer, 1908, *Hoplogryon*)

***carinifrons*** (Kieffer, 1908, *Hoplogryon*) S

***cephisus*** (Walker, 1836, *Teleas*)

***chesias*** (Walker, 1836, *Teleas*)

***chyllene*** (Walker, 1836, *Teleas*)

***elatior*** (Walker, 1836, *Teleas*) E S I

***elongatus*** (Kieffer, 1908, *Hoplogryon*) E I^^[[27]](#footnote-28)^^

***ephippium*** (Walker, 1836, *Teleas*) E I

***flavipes*** (Walker, 1836, *Teleas*) E S I

*angustula* (Thomson, 1859, *Prosacantha*)

*rufipes* (Thomson, 1859, *Prosacantha*)

*similis* (Thomson, 1859, *Prosacantha*)

*cursor* (Kieffer, 1908, *Hoplogryon*)

*tardus* (Kieffer, 1908, *Hoplogryon*)

*nigerrimus* (Kieffer, 1908, *Hoplogryon*)

*rufimanus* (Kieffer, 1908, *Hoplogryon*)

*incompletus* (Kieffer, 1908, *Hoplogryon*)

*fimbriatus* (Kieffer, 1908, *Hoplogryon*)

***galba*** (Walker, 1836, *Teleas*)

***glaucus*** (Walker, 1836, *Teleas*)

***halteratus*** (Kieffer, 1912, *Hoplogryon*)

***lamus*** (Walker, 1836, *Teleas*) I

***levigena*** (Kieffer, 1908, *Hoplogryon*) S

***lycaon*** (Walker, 1836, *Teleas*)

***marshalli*** (Kieffer, 1913, *Hoplogryon*)

***micropterus*** (Kieffer, 1908, *Hoplogryon*) S

***myrmecobius*** (Kieffer, 1911, *Hoplogryon*)

***myrmecophilus*** (Kieffer, 1911, *Paragryon*)

***nanno*** (Walker, 1836, *Gryon*)

***nitidulus*** (Thomson, 1859, *Prosacantha*) E

*pleuralis* (Kieffer, 1908, *Hoplogryon*) preocc.

*cursitans* (Kieffer, 1908, *Hoplogryon*)

*fulvimanus* (Kieffer, 1908, *Hoplogryon*)

*pleuricus* (Kieffer, 1910, *Hoplogryon*)

*bohemicus* Masner, 1962

***ocyroe*** (Walker, 1836, *Teleas*)

***opacus*** (Thomson, 1859, *Prosacantha*) I

*pedestris* misident.

***ovatus*** (Thomson, 1859, *Prosacantha*)

*orbiculatus* (Thomson, 1859, *Prosacantha*)

*petiolaris* (Thomson, 1859, *Prosacantha*)

*rotundiventris* (Thomson, 1859, *Prosacantha*)

***paula*** (Walker, 1836, *Teleas*) E W I

***procris*** (Walker, 1836, *Teleas*) E I

***punctatifrons*** (Kieffer, 1908, *Hoplogryon*) S

***puncticollis*** (Thomson, 1859, *Prosacantha*)

*hylanipennis* (Thomson, 1859, *Prosacantha*)

*coxalis* (Thomson, 1859, *Prosacantha*)

***rufonotatus*** (Kieffer, 1908, *Hoplogryon*) E S I

***sectigena*** (Kieffer, 1908, *Hoplogryon*) I added by O’Connor & Mineo (2008)

***striatigena*** (Kieffer, 1908, *Hoplogryon*) S

***therycides*** (Walker, 1836, *Teleas*) E S I

*doto* (Walker, 1836, *Teleas*)

*mermerus* (Walker, 1836, *Teleas*)

*smerdis* (Walker, 1836, *Teleas*)

*pallipes* (Thomson, 1859, *Prosacantha*)

*chloropus* (Thomson, 1859, *Prosacantha*)

*autumnalis* (Thomson, 1859, *Prosacantha*)

*brachyptera* (Thomson, 1859, *Prosacantha*)

*fuscimanus* (Kieffer, 1908, *Hoplogryon*)

*agilis* (Kieffer, 1908, *Hoplogryon*)

*microtomus* (Kieffer, 1908, *Hoplogryon*)

***timareta*** (Walker, 1836, *Teleas*) E S I

***tuberculatus*** (Kieffer, 1908, *Hoplogryon*) I added by O’Connor & Mineo (2008)

***varicornis*** (Walker, 1836, *Teleas*) E I

*metabus* (Walker, 1836, *Teleas*)

*minor* (Thomson, 1859, *Prosacantha*)

*grandis* (Thomson, 1859, *Prosacantha*)

*spinosa* (Szépligeti, 1901, *Prosacantha*)

*rufimanus* (Kieffer, 1908, *Pentacantha*)

***xenetus*** (Walker, 1836, *Teleas*)

***Xenomerus*** Walker, 1836^^[[28]](#footnote-29)^^

*NITEOGRYON* Szabó, 1966

***canariensis*** Huggert, 1974 I added by Mineo & O’Connor (2009)

*hibernicus* Mineo & O’Connor, 2009

*mutator* (Kononova & Kozlov, 2001, *Trimorus*)

***ergenna*** Walker, 1836 E I

*medon* (Walker, 1836, *Teleas*)

*curtum* (Kononova & Petrov, 1999, *Trimorus*)

Subfamily **Telenominae** Thomson, 1860

***Telenomus*** Haliday, 1833

*HEMISIUS* Westwood, 1833

*PHANURUS* Thomson, 1861

*DISSOLCUS* Ashmead, 1893

*NEONECREMNUS* Brèthes, 1909

*Allophanurus* Kieffer, 1912

*HOMOphanurus* Kieffer, 1912

*PROphanurus* Kieffer, 1912

*LIOphanurus* Kieffer, 1912

*NEOtelenomus* Dodd, 1913

*AHOLCUS* Kieffer, 1913

*NANOPRIA* Kieffer, 1913

*NEOTELEIA* Dodd, 1913

*DISSOLCOIDES* Dodd, 1913

*Platytelenomus* Dodd, 1914

*PARIDRIS* Brèthes, 1917 preocc.

*PSEUDOTELENOMUS* Costa Lima, 1928

*MICROMYMAR* Risbec, 1950

*APOROPHLEBUS* Kozlov, 1970

*PSEUDOPHANURUS* Szabó, 1975

*PSEUDOTELENOMOIDES* Szabó, 1975

*VERRUCOSICEPHALIA* Szabó, 1975^^[[29]](#footnote-30)^^

*ISSIDOTELENOMUS* Pélev, 1975

***alcon*** Walker, 1836

***andria*** Walker, 1836 I

***ater*** Haliday, 1833

***brachialis*** Haliday, 1833 W I

***cleostratus*** Walker, 1836

***coilus*** Walker, 1836 I

***colotes*** Walker, 1836

***dalmanni*** (Ratzeburg, 1844, *Teleas*) I

*orgyiae* Fitch, 1865

*fiskei* Brues, 1910

***danubialis*** (Szelényi, 1939, *Platytelenomus*) E

added by Fergusson (1983*a*)

*unilineatus* (Szabó, 1975, *Platytelenomus*)

***depressus*** (Szabó, 1975, *Verrucosicephalia*) I

added by Mineo *et al.* (2011)^^[[30]](#footnote-31)^^

***dorsennus*** Walker, 1836

***eris*** Walker, 1836 I

***fergussoni*** Buhl & O’Connor, 2012 I added by Buhl & O’Connor (2012*a*)

***heliodorus*** Mineo, 2006 I added by O’Connor & Notton (2013)

***heteropterus*** Haliday, 1833 I

*nonnitens* Szabó, 1978

*pappi* Szabó, 1978

***heydeni*** Mayr, 1879 I added by O’Connor & Mineo (2006)

***horus*** Walker, 1836

***kolbei*** Mayr, 1879 I added by Buhl & O’Connor (2011*c*)

***laeviusculus*** (Ratzeburg, 1844, *Teleas*) I added by O’Connor & Mineo (2013)

***laricis*** Walker, 1836^^[[31]](#footnote-32)^^ I

***longulus*** Kozlov, 1967 I added by O’Connor & Mineo (2009)

***lopicida*** Silvestri, 1932 I added by O’Connor & Mineo (2013)

***mentes*** Walker, 1838

***minutus*** (Westwood, 1833, *Hemisius*)

***nauplius*** Walker, 1836

***nitidulus*** (Thomson, 1861, *Phanurus*) I

*punctulatus* (Ratzeburg, 1844, *Teleas*)

*mayri* (Kieffer, 1912, *Prophanurus*)

***orphne*** Walker, 1836

***othonia*** Walker, 1836 E I

***othus*** Haliday, 1833 I

***phalaenarum*** (Nees, 1834, *Teleas*)

***phylias*** Walker, 1836 I

***pilumnus*** Walker, 1836

***punctatissimus*** (Ratzeburg, 1844, *Teleas*)

***sitius*** Walker, 1836

***stilpo*** Walker, 1836 I

***tetratomus*** (Thomson, 1861, *Phanurus*)

*bombycis* Mayr, 1879

*gracilis* Mayr, 1879

*verticillatus* Kieffer, 1917

***tritia*** Walker, 1836

***trophonius*** Walker, 1836

***truncatus*** (Nees, 1834, *Teleas*)

*linnei* (Nees, 1834, *Teleas*)

*zetterstedtii* (Ratzeburg, 1844, *Teleas*)

***turesis*** Walker, 1836 E I

*chloropus* (Thomson, 1861, *Phanurus*) Mineo *et al.* (2011)

*sokolowi* Mayr, 1897

***vibius*** Walker, 1838 E I Fergusson (1983*b*)

***vinicius*** Walker, 1836

***Trissolcus*** Ashmead, 1893

*ASOLCUS* Nakagawa, 1900

*APHANURUS* Kieffer, 1912 preocc.

*IMMSIA* Cameron, 1912

*MICROPHANURUS* Kieffer, 1926

***arminon*** (Walker, 1838, *Telenomus*) E Fergusson (1983*b*)

***belenus*** (Walker, 1836, *Telenomus*)

***cultratus*** (Mayr, 1879, *Telenomus*) E ?I added by Talamas *et al.* (2015)^^[[32]](#footnote-33)^^

*flavipes* misident.

***davatchii*** (Javahery, 1968, *Asolcus*)

***grandis*** (Thomson, 1861, *Telenomus*) E I

*nigripes* (Thomson, 1861, *Telenomus*)

*frontalis* (Thomson, 1861, *Telenomus*)

*nigrita* (Thomson, 1861, *Telenomus*)

*nixomartini* (Javahery, 1968, *Asolcus*)

*silwoodensis* (Javahery, 1968, *Asolcus*)

***theste*** (Walker, 1838, *Telenomus*)

***waloffae*** (Javahery, 1968, *Asolcus*)

Family **Sparasionidae** Dahlbom, 1858^^[[33]](#footnote-34)^^

***Sparasion*** Latreille, 1802

*OXYURUS* Lamarck, 1817 preocc.

*BEBELUS* Gistel, 1848

*PROSPARASION* Kieffer, 1913

***cephalotes*** Latreille, 1802

*frontalis* Latreille, 1805

**References**

Austin, A. D. & Field, S. A. 1997. The ovipositor system of scelionid and platygastrid wasps (Hymenoptera: Platygastroidea): comparative morphology and phylogenetic implications. *Invertebrate Taxonomy* **11**: 1-87.

Austin, A. D., Johnson, N. F. & Dowton, M. 2005. Systematics, evolution, and biology of scelionid and platygastrid wasps. *Annual Review of Entomology* **50**: 553-582.

Barnes, H. F. 1927. Material for a monograph of the British Cecidomyidae or gall midges: British gall midges of economic importance I.-V. *Journal of the South-Eastern Agricultural College* **24**: 65–146.

Barnes, H. F. 1931. Observations on gall midges affecting fruit trees. *Journal of the South-Eastern Agricultural College* **28**: 170–177.

Barnes, H. F. 1948. *Gall midges of economic importance. Vol. III: Gall midges of fruit*. Crosby Lockwood and Son Ltd, London.

Barnes, H. F. 1956. *Gall midges of economic importance. Vol. VII: Gall midges of cereal crops*. Crosby Lockwood and Son Ltd, London.

Broad, G. R. 2014. Checklist of British and Irish Hymenoptera - Introduction. *Biodiversity Data Journal* **2**: e1113. doi: 10.3897/BDJ.2.e1113

Broad, G. R. & Livermore, L. 2014*a*. Checklist of British and Irish Hymenoptera - Ceraphronoidea. *Biodiversity Data Journal* **2**: e1167.

Broad, G. R. & Livermore, L. 2014*b*. Checklist of British and Irish Hymenoptera - Evanioidea. *Biodiversity Data Journal* **2**: e1116. doi: 10.3897/BDJ.2.e1116

Buhl, P. N. 1995*a*. Taxonomic studies of *Euxestonotus* Fouts (Hym., Platygastridae). *Entomologist’s Monthly Magazine* **131**: 115–121.

Buhl, P. N. 1995*b*. Some species of Platygastridae (Hym.) new to the British List. *Entomologist’s Monthly Magazine* **131**: 122.

Buhl, P. N. 1995*c*. On two European species of *Platygaster* Latreille, 1809 (Hymenoptera Platygastridae). *Frustula Entomologica* **18**: 147-152.

Buhl, P. N. 1997. On some new or little known species of Platygastrinae (Hymenoptera, Platygastridae). *Entomofauna* **18**: 429-469.

Buhl, P. N. 2009*a*. Six new species of Platygastrinae from the British Isles (Hymenoptera, Platygastridae). *Journal of Natural History* **43**: 687–699.

Buhl, P. N. 2009*b*. Reared Palaearctic Ceraphronidae and Platygastridae (Hym.), with a new species of *Platygaster* Latreille, 1809. *Entomologist's Monthly Magazine* **145**: 197-202.

Buhl, P. N. & Bennett, F. D. 2009. Records of Platygastridae (Hym.: Platygastroidea) from the Isle of Man, with descriptions of three new species. *Entomologist’s Record and Journal of Variation* **121**: 89–99.

Buhl, P. N. & Notton, D. G. 2009. A revised catalogue of the Platygastridae of the British Isles (Hymenoptera: Platygastroidea). *Journal of Natural History* **43**: 1651-1703.

Buhl, P. N. & O’Connor, J. P. 2008. Records of Irish Platygastridae (Hymenoptera) including seventeen species new to Ireland. *Irish Naturalists’ Journal* **29**: 27–29.

Buhl, P. N. & O’Connor, J. P. 2009. 23 species of Platygastrinae (Hymenoptera, Platygastridae) new to Ireland, two of them new to science. *Irish Naturalists’ Journal* **29**: 111–115.

Buhl, P. N. & O'Connor, J. P. 2010*a*. Eleven species of Platygastrinae (Hym., Platygastridae) new to Ireland, and descriptions of the hitherto unknown males of three species. *Entomologist's Monthly Magazine* **146**: 33-35.

Buhl, P. N. & O'Connor, J. P. 2010*b*. Distributional records of Irish Platygastridae with corrections and additions to the Irish list. *Irish Biogeographical Society Bulletin* **34**: 3-17.

Buhl, P. N. & O'Connor, J. P. 2010*c*. A new species of *Synopeas* (Hym., Platygastridae) from Ireland. *Entomologist's Monthly Magazine* **146**: 195-198.

Buhl, P. N. & O'Connor, J. P. 2011*a*. A new species of *Platygaster* (Hym., Platygastridae) from Ireland. *Entomologist's Monthly Magazine* **147**: 77-80.

Buhl, P. N. & O’Connor, J. P. 2011*b*. Four species of Platygastrinae (Hym., Platygastridae) new to Ireland. *Entomologist’s Monthly Magazine* **147**: 116.

Buhl, P. N. & O'Connor, J. P. 2011*c*. Five species of Ceraphronidae and Scelionidae (Hymenoptera) new to Ireland. *Entomologist’s Monthly Magazine* **146** (2010): 154.

Buhl, P. N. & O'Connor, J. P. 2012*a*. Two new species of Platygastroidea (Hymenoptera) from Ireland. *Entomologist's Record and Journal of Variation* **124**: 195-200.

Buhl, P. N. & O'Connor, J. P. 2012*b*. Distributional records of Irish Platygastroidea: Platygastrinae and Ceraphronoidea: Ceraphronidae including *Aphanogmus elegantulus* new to Ireland. *Bulletin of the Irish Biogeographical Society* **36**: 15-23.

Buhl, P. N. & O'Connor, J. P. 2012*c*. A further four species of Platygastrinae (Hymenoptera: Platygastridae) new to Ireland. *Entomologist's Monthly Magazine* **148**: 146.

Fergusson, N. D. M. 1978. Proctotrupoida. In: Fitton, M. G., Graham, M. W. R. d. V., Bouček, Z. R. J., Fergusson, N. D. M., Huddleston, T., Quinlan, J., Richards, O. W., editors. *Kloet and Hincks. A check list of British insects. Part 4: Hymenoptera*: Handbooks for the Identification of British Insects p. 110-123.

Fergusson, N. D. M. 1983*a*. A review of the genus *Platytelenomus* Dodd (Hym., Proctotrupoidea). *Entomologist's Monthly Magazine* **119**: 199-206.

Fergusson, N. D. M. 1983*b*. The status of the genus *Allophanurus* Kieffer (Hym., Proctotrupoidea, Scelionidae). *Entomologist's Monthly Magazine* **119**: 207-209.

Fitton, M. G., Graham, M. W. R. de V., Bouček, Z. R. J., Fergusson, N. D. M., Huddleston, T., Quinlan, J. & Richards, O. W. 1978. Kloet and Hincks. A check list of British insects. Part 4: Hymenoptera. *Handbooks for the Identification of British Insects* **11(4)**: ix + 159 pp.

Gahan, A. B. 1933. The serphoid and chalcidoid parasites of the Hessian fly. *United States Department of Agriculture Miscellaneous Publications* **174**: 1–147.

Johnson, N. F. 1992. Catalog of world Proctotrupoidea excluding Platygastridae. *Memoirs of the American Entomological Institute* **51**: i-v+1-825.

Johnson, N. F. & Masner, L.. 2004. The genus *Thoron* Haliday (Hymenoptera: Scelionidae), egg-parasitoids of waterscorpions (Herniptera: Nepidae), with key to world species. *American Museum Novitates* **3452**: 1-16.

Johnson, N. F., Masner, L. & Musetti, L. 2008. Review of genera of the tribe Sparasionini (Hymenoptera: Platygastroidea, Scelionidae). *American Museum Novitates* **3629**: 1-24.

Johnson, N. F., Masner, L. & Musetti, L. 2009. *Orwellium*, a new Valdivian genus of Platygastridae (Hymenoptera). *ZooKeys* **20**: 21-30.

Kieffer, J. J. 1908. Révision des Scelionidae (Hyménoptères). *Annales de la Société Scientifique de Bruxelles* **32**: 111-250.

Kozlov, M. A. 1978. [Superfamily Proctotrupoidea]. Pp. 538-664 in: Medvedev, G. S., ed. [*Determination of insects of the European portion of the USSR*]. Leningrad: Nauka.

Liston, A. D., Knight, G. T., Sheppard, D. A., Broad, G. R. and Livermore, L. 2014. Checklist of British and Irish Hymenoptera - Sawflies, ‘Symphyta’. *Biodiversity Data Journal* **2**: e1168. doi: 10.3897/BDJ.2.e1168

Mikó, I., Masner, L. & Deans, A. R. 2010. World revision of *Xenomerus* Walker (Hymenoptera: Platygastroidea, Platygastridae). *Zootaxa* **2708**: 1-73.

Milne, D. L. 1960. The gall midges (Diptera: Cecidomyidae) of clover flower-heads. *Transactions of the Royal Entomological Society of London* **112**: 73-108 + 2 plates.

Mineo, G. & O’Connor, J. P. 2009. A new species of *Xenomerus* Walker (Hym., Scelionidae) from Ireland. *Entomologists Monthly Magazine* **145**: 97-100.

Mineo, G., O'Connor, J. P. & Ashe, P. 2011. Records of Irish scelionid wasps (Hymenoptera: Platygastroidea, Scelionidae) including notes on the genus *Verrucosicephalia* Szabó. *Irish Naturalists’ Journal* **31** (2010): 113-117.

Murchie, A. K., Polaszek, A. & Williams, I. H. 1999. *Platygaster subuliformis* (Kieffer) (Hym., Platygastridae) new to Britain, an egg-larval parasitoid of the brassica pod midge *Dasineura brassicae* Winnertz (Dipt., Cecidomyiidae). *Entomologist's Monthly Magazine* **135**: 217-222.

Murphy, N. P., Carey, D., Castro, L. R., Dowton, M. & Austin, A. D. 2007. Phylogeny of the platygastroid wasps (Hymenoptera) based on sequences from the 18S rRNA, 28S rRNA and cytochrome oxidase I genes: implications for evolution of the ovipositor system and host relationships. *Biological Journal of the Linnean Society* **91**: 653-669.

Notton, D. G. 2006. Genus-group taxa of Platygastroidea (Hymenoptera: Scelionidae and Platygastridae) new to Britain. *Entomologist’s Monthly Magazine* **142**: 189–206.

Notton, D. G. 2008. Insects of Mortimer Forest on the Shropshire/Herefordshire border. *British Journal of Entomology and Natural History* **21**: 187-192.

Notton, D. G. 2010. A catalogue of the types of Platygastridae (Hymenoptera, Platygastroidea) at the Muséum national d’Histoire naturelle, Paris, with brief notes on the history of the collection. *Zootaxa* **2358**: 1-24.

Notton, D. G., Popovici, O. A., Achterberg, C. van, De Rond, J. and Burn, J. T. 2014. Parasitoid wasps new to Britain (Hymenoptera: Platygastridae, Eurytomidae, Braconidae & Bethylidae). *European Journal of Taxonomy* **99**: 1-20.

O'Connor, J. P. & Mineo, G. 2006. Irish records of *Telenomus heydeni* Mayr and *T. othus* Haliday (Hym., Scelionidae). *Entomologist's Monthly Magazine* **142**: 184.

O'Connor, J. P. & Mineo, G. 2007. *Telenomus nitidulus* (Thomson) and *Trissolcus flavipes* (Thomson) (Hym., Scelionidae) new to Ireland. *Entomologist’s Monthly Magazine* **143**: 238.

O'Connor, J. P. & Mineo, G. 2008. *Trimorus bacilliger* (Kieffer), *Trimorus sectigena* Kieffer and *Trimorus tuberculatus* (Kieffer) (Hym., Scelionidae) new to Ireland. *Entomologist's Monthly Magazine* **144**: 30.

O'Connor, J. P. & Mineo, G. 2009. Notes on *Telenomus longulus* Kozlov and *T. laricis* Walker (Hym., Scelionidae) in Ireland. *Entomologist’s Monthly Magazine* **145**: 109.

O'Connor, J. P. & Mineo, G. 2013. *Telenomus laeviusculus* (Ratzeburg) and *Telenomus lopicida* Silvestri (Hymenoptera: Scelionidae) new to Ireland with records of other rare species. *Irish Naturalist’s Journal* **32**: 49-52.

O'Connor, J. P. & Notton, D. G. 2013. A review of the Irish scelionids (Hymenoptera: Platygastroidea, Platygastridae) including four species new to Ireland. *Bulletin of the Irish Biogeographical Society* **37**: 20-44.

O’Connor, J. P., Nash, R., Notton, D. G. & Fergusson, N. D. M. 2004. A catalogue of the Irish Platygastroidea and Proctotrupoidea (Hymenoptera). *Occasional Publications of the Irish Biogeographical Society* **7**: 110 pp.

Orr, D. B. 1988. Scelionid wasps as biological control agents: a review. *Florida Entomologist* **71**: 506-528.

Polaszek, A. 1997. *Amitus* Haldeman (Hymenoptera: Platygastridae): a genus of whitefly parasitoids new to Britain. *Entomologist's Monthly Magazine* **133**: 77-79.

Popovici, M. and Buhl, P. N. 2010. The West Palaearctic species of *Fidiobia* Ashmead, 1894 (Hymenoptera: Platygastroidea). *Journal of Natural History* **44**: 1131-1164.

Sharkey, M. J. 2007. Phylogeny and classification of Hymenoptera. *Zootaxa* **1668**: 521-548.

Talamas, E. J., Johnson, N. F., Van Noort, S., Masner, L. & Polaszek, A. 2009. Revision of world species of the genus *Oreiscelio* Kieffer (Hymenoptera, Platygastroidea, Platygastridae). *ZooKeys* **6**: 1-68.

Talamas, E. J., Johnson, N. F. and Buffington, M. 2015. Key to Nearctic species of *Trissolcus* Ashmead (Hymenoptera, Scelionidae), natural enemies of native and invasive stink bugs (Hemiptera, Pentatomidae). *Journal of Hymenoptera Research* **43**: 45-110.

Vlug, H. J. 1995. *Catalogue of the Platygastridae (Platygastroidea) of the* *world*. In: van Achterberg, C., ed. Hymenopterorum Catalogus **19**. Amsterdam: SPB Academic Publishing. 168 pp.

1. Buhl & Notton (2009) revised the British and Irish checklist of Platygastrinae and Sceliotrachelinae (i.e. the traditional Platygastridae) and this forms the basis for the nomenclature and distribution data recorded here. Additional records from Buhl & O’Connor (2010*a*,*b*, 2011*b*, 2012*b*,*c*) and a few from PB. Dates of description and original combinations follow Vlug (1995). [↑](#footnote-ref-2)
2. PB regards *rufopetiolata* as a valid species, not a subspecies of *lasiophila*, although he has not seen the type. [↑](#footnote-ref-3)
3. British specimens have been assigned to the subspecies *hyalina* Kieffer, 1914 (Buhl & Notton, 2009). [↑](#footnote-ref-4)
4. Overlooked by Fergusson (1978) and by Buhl & Notton (2009). [↑](#footnote-ref-5)
5. Buhl & Notton (2009) record specimens tentatively identified as *hyperici* from England and Scotland. [↑](#footnote-ref-6)
6. Omitted by Fergusson (1978). [↑](#footnote-ref-7)
7. Recorded by Buhl & Notton (2009) as *Isostasius inserens sensu* Kozlov, 1978, not *inserens* (Kirby, 1800). [↑](#footnote-ref-8)
8. NHM, det. PB. [↑](#footnote-ref-9)
9. Omitted by Fergusson (1978). [↑](#footnote-ref-10)
10. Omitted by Fergusson (1978). [↑](#footnote-ref-11)
11. Omitted by Fergusson (1978). [↑](#footnote-ref-12)
12. Omitted by Fergusson (1978). [↑](#footnote-ref-13)
13. Tentative identification (based on Scottish material) by Buhl & Notton (2009) but recorded, from reared English specimens, without any question marks by Buhl (2009*b*). [↑](#footnote-ref-14)
14. Tentatively identified individuals recorded from Wales too (Buhl & Notton, 2009). [↑](#footnote-ref-15)
15. English record from Buhl (1995*c*). [↑](#footnote-ref-16)
16. NHM, det. PB. [↑](#footnote-ref-17)
17. Listed as *Synopeas* cf. *convexum*, occurring in England and Ireland, by Buhl & Notton (2009), then as a certain identification of Irish specimens by Buhl & O’Connor (2010*a*). [↑](#footnote-ref-18)
18. *Synopeas muticus* (Nees, 1834, *Platygaster*), as recorded by Buhl & O’Connor (2008) refers to *sosis* (Buhl & O’Connor, 2010*b*). [↑](#footnote-ref-19)
19. Described from English specimens, omitted by Fergusson (1978). [↑](#footnote-ref-20)
20. *Amitus minervae* auctt., not *A. minervae* Silvestri. [↑](#footnote-ref-21)
21. Recorded as *Fidiobia synergorum* (Kieffer, 1921, *Fahringeria*) by O’Connor *et al.* (2004). [↑](#footnote-ref-22)
22. Some distribution records from O’Connor & Notton (2013). [↑](#footnote-ref-23)
23. Transferred from *Opisthacantha* by Notton (2006), who provides distribution data. [↑](#footnote-ref-24)
24. Transferred from *Trimorus* by Notton (2006), who provides distribution data. [↑](#footnote-ref-25)
25. Distribution and synonymy from Johnson & Masner (2004). [↑](#footnote-ref-26)
26. Although described from Scotland by Kieffer (1908), listed as a synonym of *Trimorus pedestris* (Nees) (now placed in *Gryon*) by Fergusson (1978) until raised from synonymy by O’Connor & Mineo (2008). [↑](#footnote-ref-27)
27. Although described from probable English material by Kieffer (1908), listed as a synonym of *puncticollis* by Fergusson (1978) but raised from synonymy by Kozlov (1978). [↑](#footnote-ref-28)
28. Distribution data and synonymy from Mikó *et al.* (2010). [↑](#footnote-ref-29)
29. Mineo *et al.* (2011) argue that *Verrucosicephalia* should be recognised as a separate genus, although N. Johnson *et al.*, in the online Platygastroidea catalogue (<http://osuc.biosci.ohio-state.edu/hymDB/eol_scelionidae.home>) retain the name as a synonym of *Telenomus*. [↑](#footnote-ref-30)
30. As a species of *Verrucosicephalia*. [↑](#footnote-ref-31)
31. Listed by Mineo *et al.* (2011) as a species of *Verrucosicephalia*. [↑](#footnote-ref-32)
32. The Irish record of *Trissolcus flavipes* (Thomson, 1860, *Telenomus*) by O'Connor and Mineo (2007) probably refers to *cultratus*(Talamas *et al*. 2015). [↑](#footnote-ref-33)
33. Nomenclature follows Johnson *et al.* (2008). [↑](#footnote-ref-34)
